# Molecular Epidemiological Characterization of *Staphylococcus argenteus* Clinical Isolates in Japan: Identification of Three Clones (ST1223, ST2198, and ST2550) and a Novel Staphylocoagulase Genotype XV

**DOI:** 10.3390/microorganisms7100389

**Published:** 2019-09-24

**Authors:** Meiji Soe Aung, Noriko Urushibara, Mitsuyo Kawaguchiya, Ayako Sumi, Seika Takahashi, Miyo Ike, Masahiko Ito, Satoshi Habadera, Nobumichi Kobayashi

**Affiliations:** 1 Department of Hygiene, Sapporo Medical University School of Medicine, Hokkaido, Sapporo 060-8556, Japan; noriko-u@sapmed.ac.jp (N.U.); kawaguchiya@sapmed.ac.jp (M.K.); sumi@sapmed.ac.jp (A.S.); nkobayas@sapmed.ac.jp (N.K.); 2 Sapporo Clinical Laboratory, Inc., Hokkaido, Sapporo 060-0005, Japan; tkhs_seika@yahoo.co.jp (S.T.); saturin-saikin@saturin.co.jp (M.I.); m-ito@saturin.co.jp (M.I.); s-habadera@saturin.co.jp (S.H.)

**Keywords:** *Staphylococcus argenteus*, ST, staphyocoagulase genotype, enterotoxin (-like) genes, Japan

## Abstract

*Staphylococcus argenteus*, a novel emerging species within *Staphylococcus aureus* complex (SAC), has been increasingly reported worldwide. In this study, prevalence of *S. argenteus* among human clinical isolates, and their clonal diversity and genetic characteristics of virulence factors were investigated in Hokkaido, the northern main island of Japan. During a four-month period starting from March 2019, twenty-four *S. argenteus* and 4330 *S. aureus* isolates were recovered from clinical specimens (the ratio of *S. argenteus* to *S. aureus* :0.0055). Half of *S. argenteus* isolates (*n* = 12) belonged to MLST sequence type (ST) 2250 and its single-locus variant, with staphylocoagulase genotype (*coa*-) XId, while the remaining isolates were assigned to ST2198/*coa*-XIV (*n* = 6), and ST1223 with a novel *coa*-XV identified in this study (*n* = 6). All the isolates were *mecA*-negative, and susceptible to all the antimicrobials tested, except for an ST2198 isolate with *blaZ* and an ST2250 isolate with *tet(L)* showing resistance to ampicillin and tetracyclines, respectively. Common virulence factors in the *S. argenteus* isolates were staphylococcal enterotoxin (-like) genes *sey*, *selz*, *sel26*, and *sel27* in ST2250, *selx* in ST2198, and enterotoxin gene cluster (*egc-1*: *seg-sei-sem-sen-seo*) in ST1223 isolates, in addition to hemolysin genes (*hla, hlb,* and *hld*) distributed universally. Elastin binding protein gene (*ebpS*) and MSCRAMM family adhesin SdrE gene (*sdrE*) detected in all the isolates showed high sequence identity among them (> 97%), while relatively lower identity to those of *S. aureus* (78–92%). Phylogenetically, *ebpS*, *sdrE*, *selx*, *sey*, *selw*, *sel26*, and *sel27* of *S. argenteus* formed clusters distinct from those of *S. aureus*, unlike *sec*, *selz*, *tst-1*, and staphylokinase gene (*sak*). The present study revealed the prevalence of *S. argenteus* among clinical isolates, and presence of three distinct *S. argenteus* clones (ST2250; ST2198 and ST1223) harboring different virulence factors in northern Japan. ST2198 *S. argenteus*, a minor clone (strain BN75-like) that had been rarely reported, was first identified in Japan as human isolates.

## 1. Introduction

*Staphylococcus argenteus* is a novel species of coagulase-positive staphylococci previously described as a divergent lineage of *S. aureus* [1], and has been increasingly reported worldwide as an emerging pathogen affecting both humans [2,3,4,5,6,7,8,9,10,11,12,13,14,15,16,17,18,19,20,21,22,23,24,25,26] and animals [1,25,27,28,29]. The major characteristic of this lineage is non-pigmented (white) colonies on blood agar plates due to lack of the *crtOPQMN* gene operon required for staphyloxanthin pigment production [1,2]. *S. argenteus* and *S. aureus* are unable to be discriminated by routine diagnostic microbiological testing, and also by analysis of 16S rRNA gene, because these species have identical sequence of this gene [1]. However, *S. argenteus* shows relatively low genome sequence identity (87%) to *S. aureus*, and only 4% genes were shared between the two species at 100% homology level [1,3]. Accordingly, evident sequence difference was documented in early reports for the thermonuclease gene (*nuc*) and the nonribosomal peptide synthetase (*NRPS*) gene [1,2,4], which are available as markers for identification of these species. Thereafter, sequence diversity and genetic characteristics between these species were revealed for genes encoding major virulence factors, i.e., staphylococagulase, protein A, and alpha-hemolysin [5].

The type strain of *S. argenteus* MSHR1132^T^ belongs to ST1850 grouped into clonal complex 75 (CC75) [1]. After the first report of genetically divergent ST75 in Australia in 2002 [6,7], *S. argenteus* belonging to CC75 and other CC represented by CC1223, CC2198, CC2250, and CC2596 were increasingly reported in other Oceanian countries (New Zealand [8] and Fiji [8,9]), Asia (Thailand [10,11], Lao PDR [12], Cambodia [13], Myanmar [5,14], China [4], Japan [15,16,17] and Taiwan [18]), Europe (Belgium [19], France [20], the UK [10], Denmark [3] and Sweden [21]) and South America (French Guiana [22] and Trinidad and Tobago [23]). There are at least three geographical ‘hot spots’ of *S. argenteus*, such as Southeast Asia, remote human populations in Australia and the Amazon [24,25]. *S. argenteus* infections in humans have been commonly community-onset [11,18], occasionally associated with high mortality [18], and some isolates were revealed to harbor Panton-Valentine leucocidin that may be related to severe diseases in humans [14,20]. However, information on *S. argenteus* reported to date are still insufficient to delineate its epidemiology, clinical significance and nosocomial impact, containing partly contradictory views [25]. In Japan, only limited information of *S. argenteus* in humans are available; a case report of purulent lymphadenitis [26], two reports of food poisoning outbreaks [15,16], and two cases of bacteremia through retrospective study [17], while the prevalence of *S. argenteus* among presumptive *S. aureus* from general clinical specimens has not yet clearly been understood.

In the present study, we investigated the prevalence of *S. argenteus* from various clinical specimens from patients in Hokkaido, the northern main island of Japan, and analyzed their genotypes and virulence factors including toxins and adhesins. The results indicated the presence of three clones with different toxin gene profiles including newer enterotoxin-like genes (*selz*, *sel26*, and *sel27*), novel staphylocoagulase genotype XV, and *S. argenteus*-specific genetic groups in the primary virulence factors (SdrE, elastin-binding protein).

## 2. Materials and Methods

### 2.1. Bacterial Isolates, Species Identification

*S. aureus* and *S. argenteus* were isolated from various clinical specimens that were brought to the Sapporo Clinical Laboratory Inc., Sapporo, Japan, from both hospitalized patients and outpatients in medical facilities in Hokkaido, during a four-month period starting on 7th March 2019. The clinical specimens were inoculated onto blood agar plates and incubated at 37 °C for 24 h aerobically. Gram-positive, coagulase-positive isolates were collected for further bacteriological identification. Initial screening of *S. argenteus* was performed by MALDI-TOF mass spectrometry using MALDI Biotyper (BRUKER). Isolates assigned as *S.argenteus* were confirmed genetically by three methods: (1) PCR targeting *crtOPQMN* gene operon-deficient region, (2) PCR targeting *NRPS* [4], and (3) sequence analysis of the partial *NRPS* gene [4] and *nuc* gene [1]. The method (1) was designed in the present study and performed by multiplex PCR to amplify *crtOPQMN*-deficient region (*S. argenteus*) or *crtP* (*S. aureus*) using primers listed in Table S1. In the method (2), non-*S. aureus* SAC (*S. argenteus*/*S. schweitzeri*)-specific amplicon that is 180-bp longer than that of *S. aureus* was detected. In method (3), full-length nucleotide sequence of *nuc* and partial *NRPS* gene were determined by PCR and direct sequencing, followed by alignment with those of *S. aureus* and *S. argenteus* using Clustal Omega program (https://www.ebi.ac.uk/Tools/msa/clustalo/).

### 2.2. Antimicrobial Susceptibility Testing

Minimum inhibitory concentrations (MICs) within limited ranges were measured by broth microdilution test against 18 antimicrobial agents (oxacillin, OXA; ampicillin, AMP; cefazolin, CFZ; cefmetazole, CMZ; flomoxef, FMX; imipenem, IPM; gentamicin, GEN; arbekacin, ABK; erythromycin, ERY; clindamycin, CLI; vancomycin, VAN; teicoplanin, TEC; linezolid, LZD; minocycline, MIN; Fosfomycin, FOF; levofloxacin, LVX; cefoxitin, FOX; trimethoprim/sulfamethoxazole, SXT) by using the Dry Plate ‘Eiken’ DP32 (Eiken Chemical, Tokyo, Japan), and also tetracycline and doxycycline manually. Resistance or susceptibility was judged according to the breakpoints defined in the Clinical Laboratory Standards Institute (CLSI) guidelines [30] for most of the antimicrobial drugs examined. For fosfomycin and arbekacin, whose break points are not defined by CLSI guidelines, the European Committee on Antimicrobial Susceptibility Testing (EUCAST) breakpoint (FOF, 32 μg/mL, *Staphylococcus* spp.) [31] and a unique breakpoint (ABK, 4μg/mL, which is higher than the 2 μg/mL defined by the Japanese Society of Chemotherapy for respiratory infection) were used [32]. A breakpoint of flomoxef (16 μg/mL) defined by the Japanese Society of Chemotherapy for urinary tract infection was also applied [32].

### 2.3. Genetic Typing, Detection of Virulence Factors and Drug Resistance Genes

For all the isolates, presence of staphylococcal 16S rRNA, *nuc*, *mecA*, PVL genes and ACME-*arcA* (arginine deiminase gene) was examined by multiplex PCR assay as described by Zhang et al. [33]. Sequence type (ST) was determined according to the scheme of multilocus sequencence typing (MLST) (https://pubmlst.org/) [34]. *spa* type based on sequence of protein A gene X-region was determined by PCR and sequencing as described previously [35] using Ridom SpaServer (http://spa.ridom.de/index.shtml). Staphylocoagulase genotype (*coa*-type) was determined by multiplex PCR assay as described previously [36]. For the isolates of which *coa*-type was not classified by the multiplex PCR, partial staphylocoagulase gene sequences (D1, D2, and the central regions) were determined and their highly similar staphylocoagulase sequences were searched by Basic Local Alignment Search Tool (BLAST: https://blast.ncbi.nlm.nih.gov/Blast.cgi) to assign their *coa*-types. For untypeable isolates, nucleotide sequences of whole staphylocoagulase gene was determined as described previously [37], subsequently sequence identities of D1 region, D2 and the central (C) region to those of established *coa*-types were analyzed by using Clustal Omega program.

The presence of 28 staphylococcal enterotoxin (SE) (-like) genes (*sea-see*, *seg-selu*, *selx*, *sey*, *selw*, *selz*, *sel26* and *sel27*), the TSST-1 gene (*tst-1*) and exfoliative toxin genes (*eta*, *etb* and *etd*), leukocidins (*lukDE* and *lukM*), haemolysins (*hla*, *hlb*, *hld* and *hlg*), adhesin genes (*eno*, *cna*, *sdrC*, *sdrD*, *sdrE*, *fib*, *clfA*, *clfB*, *fnbA*, *fnbB*, *icaA*, *icaD*, *ednA*, *ednB*, *bap* and *vWbp*), modulators of host defense (*sak*, *chp* and *scn*) were analyzed by multiplex or uniplex PCRs [38,39,40]. For detection of *selz*, *sel26*, and *sel27* by PCR, primers shown in Table S1 were newly designed based on *selz* sequence reported for bovine *S. aureus* strain RF122 (GenBank accession no. NC_007622, locus tag SAB_RS00140) [41] and *sel26/sel27* sequences reported for *S. aureus* strain 50 and RF14 (GenBank accession no. MF370874 and CP011528, respectively) [42]. Terms of “*sel27*” and “*sel28*” assigned to strain 50 in the annotations in GenBank database were identical to “*sel26*” and “*sel27*”, respectively, described by Zhang and coworkers [42].

Genes conferring resistance to penicillin (blaZ), tetracycline (tet(K), tet(L), and tet(M)), macrolide (ermA, ermB, ermC, and msrA), aminoglycoside (aac(6′)-Im, aac(6′)-Ie-aph(2”)-Ia, ant(3”)-Ia, ant(4′)-Ia, ant(6)-Ia, ant(9)-Ia, ant(9)-Ib, aph(2”)-Ib, aph(2”)-Ic, aph(2”)-Id, and aph(3′)-IIIa) were detected by uniplex or multiplex PCR using the primers previously reported [38,39,40].

### 2.4. Phylogenetic Analysis of Virulence Factors

Nucleotide sequences of full-length ORF were determined for staphylocoagulase genes belonging to a novel type, microbial surface component recognizing adhesive matrix molecule (MSCRAMM) family adhesin SdrE gene (*sdrE*), elastin-binding protein gene (*ebpS*), SE(-like) genes (*selw*, *selx*, *sey*, *selz*, *sel26*, *sel27*), and staphylokinase gene (*sak*) by PCR with primers listed in Table S1, followed by Sanger sequencing using the BigDye Terminator v3.1 Cycle Sequencing kit (Applied Biosystems, Foster City, CA, USA) on an automated DNA sequencer (ABI PRISM 3100). Similarly, full length sequences of enterotoxin gene *sec* and *tst-1* gene were also determined. Phylogenetic dendrograms of these virulence factors were constructed by the maximum likelihood method using the MEGA7 software, together with sequence data of staphylococcal strains available in GenBank database. The dendrograms were statistically supported by bootstrapping with 1000 replicates. Clustal Omega program was used for multiple alignments of amino acid sequences of EbpS. Prediction of transmembrane/hydrophobic region in EbpS was performed by TMpred program (https://embnet.vital-it.ch/software/TMPRED_form.html). All the sequence data of *S. argenteus* genes (*nuc*, *nrps*, *sec*, *selx*, *sey*, *selw*, *selz*, *sel26*, *sel27*, *tst-1*, *sdrE*, *ebpS* and *sak*) determined in the present study were deposited in GenBank database under accession numbers shown in Table S2.

## 3. Results

### 3.1. Identification and Prevalence of S. argenteus

A total of twenty-four *S. argenteus* isolates derived from twenty-three patients (15 outpatients and 8 inpatients) were isolated as listed in Table 1. From an isolate of a single patient (ear discharge), two clones showing hemolysis on blood agar plate (SG05-1) and no hemolysis (SG05-2) were isolated. From an inpatient, two isolates (SG22, SG23) were recovered from different specimens (pharynx and blood, respectively). *S. argenteus* isolates were derived from various specimens, with those from respiratory system being dominant (sputum (*n* = 6), pharynx (*n* = 2), nasal discharge (*n* = 2)), followed by stool (*n* = 4), skin and abscess (*n* = 3), urine (*n* = 2), vaginal discharge (*n* = 2), ear discharge (*n* = 1), blood (*n* = 1), and subdural abscess (*n* = 1). Specimens were brought from eleven cities/towns, and ratio of outpatients to inpatient was 5:3. Age range of the patient was 14–94 years, with equal sex ratio. During the same study period, number of non-duplicate *S. aureus* isolates was 4330, that comprised 2817 methicillin-susceptible *S. aureus* (MSSA) and 1513 methicillin-resistant *S. aureus* (MRSA). Isolation ratios of *S. argenteus* to *S. aureus*, and to MSSA were 0.0055 (0.55%, 24/4330) and 0.0085 (0.85%, 24/2817), respectively.

Thermostable nuclease gene (*nuc*) of all the 24 isolates comprised 669 nucleotides that was shorter than that of *S. aureus* by 18 bp, and showed 98.8–100% identity to those of *S. argenteus* strains (MSHR1132^T^, BN75, XNO62, XNO106, 51183) (Table S3), while 82% to *S. aureus nuc* gene. Similarly, partial *NRPS* gene sequences (264 bp) of the 24 isolates had 98.1–100% identity among them and to those of the reported *S. argenteus* strains, in contrast they were 180-bp longer than that of *S. aureus* strain NCTC8325 showing 60–61% identity.

### 3.2. Genotypes (ST, spa Type, coa-Type)

Among the twenty-four *S. argenteus* isolates, half of them (*n* = 12) was classified into ST2250 (11 isolates) and its single-locus variant (ST3951, one isolate), and remainings were assigned to ST2198 (25%, *n* = 6), and ST1223 (25%, *n* = 6) (Table 1). ST2250/ST3951 isolates were classified into *spa* type t5078 (6 isolates) and other *spa* types (t5787, t7960, t17928, and unassigned types) having similar repeat profile to t5078. ST2198 isolates were assigned to t7959 or other types with t7959-like profile, while ST1223 isolates were typed as t5142 or t5142-related types (Table 1).

Because *coa*-type of *S. argenteus* isolates could not be assigned to I through X by the multiplex PCR method, sequence identities of D1 region and D2-C region to those of established *coa*-types Ia-XIV were analyzed. All the ST2250 isolates were assigned to staphylocoagulase genotype XId, while ST2198 isolates to *coa*-XIV.

According to the criteria to define *coa*-type proposed previously [43], staphylocoagulase genes showing > 90% sequence identity in D1 region and D2-C regions are assigned to identical *coa*-type and subtype within the *coa*-type, respectively. D1 and D2-C regions of six isolates (SG03, SG06, SG07, SG15, SG17 and SG18) showed less than 88.3% and 82.9% identities to the 14 established *coa*-types, respectively, while 100% identities to *S. argenteus* strain SJTU F20214, D7903 and M051_MSHR of which *coa*-type has not yet been described (Table 2). Therefore, a novel staphylocoagulase genotype XV was created and assigned to all the six ST1223 *S. argenteus* isolates.

### 3.3. Prevalence of Virulence Factors, Drug Resistance Genes and Antimicrobial Susceptibility

None of the isolates had PVL genes (*lukS-PV-lukF-PV*) or ACME-*arcA* genes. Alpha-, beta-, and delta-hemolysin genes (*hla*, *hlb*, *hld*) were detected in all the isolates (Table 1). SE-like genes *sey*, *selz*, *sel26* and *sel27* were detected in mainly ST2250 isolates, while *selz* was harbored by some ST2198 and ST2113 isolates. *selx* was found in all the ST2198 isolates and one isolate each of ST2250 and ST1223. All the ST1223 isolates harbored *seg-sei-sem-sen-seo* (*egc-1*) and *selw*. *sec* and *tst-1* genes were detected in three and two isolates of ST2250, respectively. However, all other remaining SE genes and exfoliative toxin genes examined were not detected. Staphylokinase gene (*sak*) was detected in seven isolates (six ST2250 and one ST2198 isolates). Nine adhesin genes (*fib*, *clfA*, *clfB*, *ebpS*, *eno*, *fnbA*, *fnbB*, *sdrE*, *icaA*) were identified in all the isolates, while most isolates had *sdrC* and *sdrD*. Two hemolysis-positive and negative clones (SG05-1 and SG05-2, respectively) from a patient, were classified into the same genotypes (ST2250/*coa*-XId) and showed identical profiles of virulence factors.

All the isolates were methicillin-sensitive without having *mecA*, and susceptible to 20 antimicrobials tested, except for two isolates; an ST2198 isolate SG20 with *blaZ* was resistant to ampicillin (MIC, 4 μg/mL) and an ST2250 isolate SG23 with *tet(L)* showed resistance to tetracycline (MIC, 64 μg/mL) and intermediate resistance to doxycycline (MIC, 8 μg/mL). None of the isolates harbored other resistance genes tested.

### 3.4. Phylogenetic Analysis of Virulence Factors

**Figure 1:**
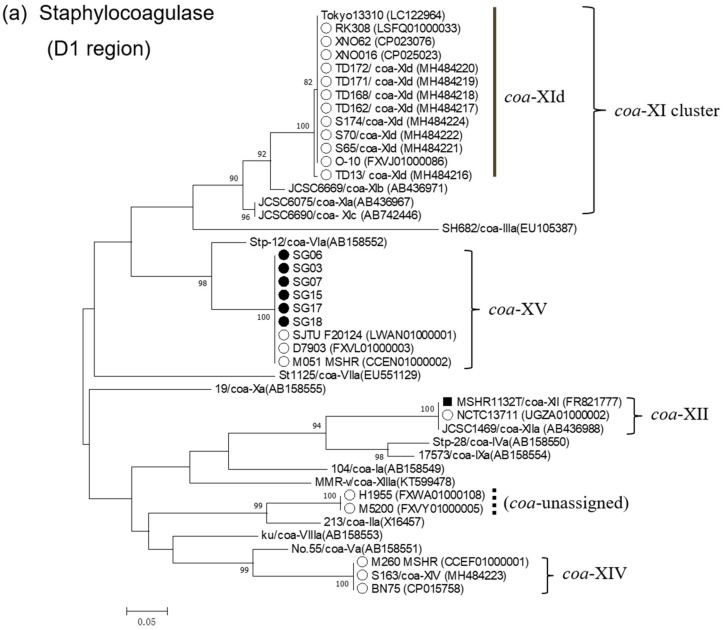

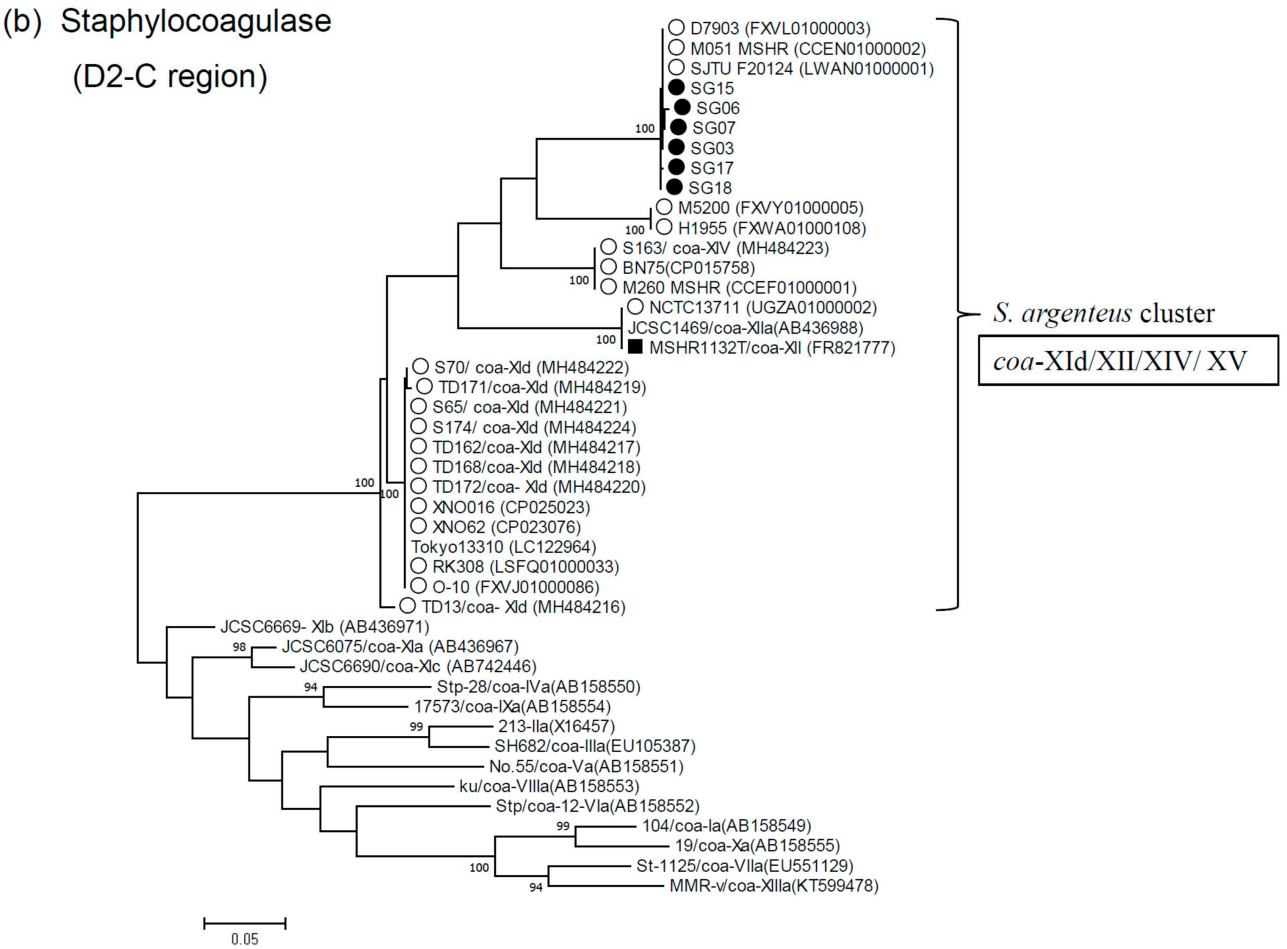
Phylogenetic dendrograms of staphylocoagulase gene encoding D1 region (**a**) and D2-C region (**b**) of *S. argenteus* and *S. aureus* strains constructed by maximum-likelihood method with MEGA.7 program. Trees were statistically supported by bootstrapping with 1000 replicates, and genetic distances were calculated by Kimura two-parameter model. Variation scale is described at the bottom. Percent bootstrap support is indicated by the values at each node (the values < 80 are omitted). Filled circle and open circle indicate *S. argenteus* isolates isolated in the present study and those reported elsewhere previously, respectively. A square indicates strain MSHR1132^T^. Others are *S. aureus* strains representing individual coagulase genotypes. Clusters of *coa*-XI, -XII, -XIV, and -XV (**a**) and *S. argenteus* cluster (**b**) are shown on the right.

To clarify phylogenetic relatedness of staphylocoagulase genes of the novel *coa*-type XV to those of other types, dendrograms of D1 and D2-C regions of staphylocoagulase genes were constructed (Figure 1a,b, respectively). In D1 region, *S. argenteus* strains were discriminated into five clusters representing *coa*-XId, -XII, -XIV, -XV, and an unassigned type. *coa*-XV D1 region was revealed to be of the same lineage as that of *coa*-VIa, although sequence identity was 88.3% (Table 2). In contrast, D2-C regions of all the *S. argenteus* strains including *coa*-XV clustered in a single phylogenetic group, which was distinctive from those of *S. aureus*.

SE-like toxin genes *selx*, *selw*, and *sel27* in *S argenteus* isolates, were phylogenetically distinct from those of *S. aureus* with 94.4–98.4% sequence identity, forming isolated clusters (Figure S1). Similarly, *sey* and *sel26* of *S. argenteus* formed clusters evidently distinct from *S. aureus*, while showing 98–99% identity to those of *S. aureus*. In contrast, *selz* of *S. argenteus* was not distinctive from that of *S. aureus*, likewise *sec*, *tst-1* and *sak* having 98–100% sequence identity to *S. aureus*. (Table S4)

Elastin binding protein gene (*ebpS*) was phylogenetically distinct between *S. argenteus* and *S. aureus* with only 78–89% identity, while higher sequence identity (>98.9%) was noted among *S. argenteus* isolates. Despite the lower identity and difference in amino acid length, *S. argenteus* EbpS contained three hydrophobic domains (H1, H2, and H3) that were located at almost the same positions as in the *S. aureus* EbpS [44], among which H3 domain was the most conserved between the two species (Figure S2).

SdrE genes (*sdrE*) was revealed to have two major lineages I and II, both of which contained *S. argenteus* clusters (cluster 1 and 2, respectively). ST2250 and ST1223 isolates were grouped into cluster 1, which was closely related to an *S. schweitzeri* strain, while cluster 2 comprised ST2198 *S. argenteus* including strains MSHR1132^T^ and BN75. Within a same cluster, *S. argenteus sdrE* showed >97% identity, while 88.6–90.2% identity between cluster 1 and 2. Within a same lineage, identity of *sdrE* sequence between *S. aureus* and *S. argenteus* was 90–92.7%. Amino acid sequence alignment of SdrE revealed that TYTFTDYVD motif, which is conserved within MSCRAMMs (ClfA, ClfB, SdrC, SdrD, SdrE) of *S. aureus* [45], are also found in *S. argenteus* isolates, although only lineage II (ST2198) strains had a modified motif (TYKFTDYVD, single amino acid difference is underlined) that was reported for Bbp (bone sialoprotein-binding protein, an allelic variant of SdrE) [46] (Figure S3). In addition, CnaBE3 domain, which is implicated in immunological protection of bacterial cells in mouse model [47] was also highly conserved, except for an ST1223 isolate SG06.

## 4. Discussion

Several clonal complexes including more than 60 STs of *S. argenteus* have been described so far [1,3], and some CCs (CC75 and CC2250) show a wide geographical distribution indicating international spread [48]. The prevalence of *S. argenteus* reported to date have varied depending on geographical areas, study populations and infection sites. In northern Australia, CC75 *S. argenteus* accounted for 8% of MSSA and 69% of MRSA isolated from skin infections of indigenous children [1].

Two separate studies in Thailand revealed an *S. argenteus* prevalence of 19% for community acquired MRSA causing sepsis [11] and 4.1% among invasive staphylococcal infections [10]. In Lao PDR, 6% of presumptive *S. aureus* isolates from skin and soft tissue infections were identified as *S. argenteus* [12]. In Myanmar, prevalence of *S. argenteus* was reported as 0.9% (5/563) from healthy nasal carriers [14] and 2.9% (4/137) [5] in clinical isolates. In contrast, the prevalence seems to be low in European countries (< 1%, Belgium (0.16%) [19], Sweden (0.3%) [21] and Denmark (0.35%) [3], and eastern China (0.7%) [4]. In our present study, the prevalence (ratio of *S. argenteus* to *S. aureus*) in the northern Japan was revealed to be 0.55% (0.85% to MSSA), that was lower than Southeast Asian countries but comparable to those of European countries and eastern China. The 24 *S. argenteus* isolates in our study were derived from 11 cities/towns, and various specimens from inpatients and outpatients of all age groups, suggesting that this species may be widely distributed to general population endemically, despite considerably lower prevalence than *S. aureus*.

Among *S. argenteus*, ST2250 has been described as the most widely spread genotype [45], and found to be a dominant clone in Thailand [10,11,24], Denmark [3], and Myanmar [5,14]. In contrast, ST1223 (CC1223) and ST2198 (CC2198) are recognized as minor lineages [3,45], and reported in Thailand [10,11] and Lao PDR [12] at low detection rate. In Japan, food poisoning outbreaks were caused by ST1223 *S. argenteus* strains [15,16], while a purulent lymphadenitis and bacteremia cases by ST2250 strain [17,26]. Despite the dominance of ST2550, our present surveillance suggested that ST1223 *S. argenteus* may not be a rare clone in Japan. All the six ST1223 isolates in our study had enterotoxin gene cluster (*egc-1*), as reported for isolates from the food poisoning [15], and three and one isolates were derived from stools and subdural abscess, respectively. Thus, it is suggested that ST1223 may be related to gastrointestinal and/or invasive infections. Meanwhile, *S. argenteus* of ST2198 was first reported in Japan in our study. Genetically, most well-characterized ST2198 strain is BN75 which was isolated from feces of a western lowland gorilla in Gabon [27]. This strain has less virulence factor genes and no drug resistance genes, showing susceptibility to all the antimicrobials tested, which was similar characteristics to our present ST2198 isolates.

Along with *coa*-XII that was assigned to strain MSHR1132^T^, *S. argenteus* strains reported to date belong to at least four *coa*-types, XId, XII, XIV, and XV, which are scarcely detected in *S. aureus*. The *coa*-type is defined by sequence diversity in the D1 and D2 regions, which contain antigenic regions and are associated with prothrombin-binding of staphylocoagulase [49]. Therefore, the presence of the *coa*-types in *S. argenteus* distinctive from *S. aureus* suggests that *S. argenteus* staphylocoagulase might have evolved through selective pressure with antibodies and/or prothrombin in animal species that is different from humans. This assumption is supported by the finding of *S. argenteus* isolates in Thailand which are genetically related to livestock-associated *S. aureus* [24].

In this study, prevalence of SE (-like) genes in *S. argenteus* isolates was revealed to be generally different depending on the clone. A report in Thailand described that *S. argenteus* has less virulence factors compared with *S. aureus*, suggesting its less virulence to humans [11]. In contrast, higher risk of mortality was reported for *S. argenteus* bacteremia than that of *S. aureus* [18]. Although it is not certain whether toxins are sole determinants of virulence, it is evident that prevalence of virulence factors is different depending on *S. argenteus* clone. Thus, virulence of *S. argenteus* is suggested to be variable by individual clones. Because *S. argenteus* genome has 87% identity to that of *S. aureus* [1], and as high as 38% of *S. argenteus* genes have only 85% homology to *S. aureus* [3], it is conceivable that *S. argenteus* may have any unidentified virulence factors, or variants of virulence factors commonly shared with *S. aureus*.

Phylogenetic analysis revealed that SE (-like) genes, *selw*, *selx*, *sey*, *sel26* and *sel27* of *S. argenteus* were genetically distinguished from those in *S. aureus*, despite showing high sequence identity to *S. aureus*, suggesting that these genes might have been shared among the two species or present in the most recent common ancestor since long time ago, followed by evolving within individual species. Similar findings were reported for protein A and alpha-hemolysin genes in our previous study [5]. In contrast, *sec*, *selz*, *tst-1* and *sak* detected in *S. argenteus* isolates were almost identical to those of *S. aureus*, indicating that dissemination of these genes might have occurred more recently.

It was notable in the present study that *ebpS* and *sdrE* genes were genetically distinct from those of *S. aureus* with lower identity for *ebpS* (78–89%) and slightly high identity for *sdrE* (90–92%). However, elastin binding protein (EbpS) of *S. argenteus* was presumed to have three hydrophobic domains at the same positions as *S. aureus* [44], suggesting that it has the same topology and function as *S. aureus* EbpS. Similarly, TYTFTDYVD motif and CnaBE3 domain of SdrE were conserved among *S. argenteus* and *S. aureus*, suggesting that SdrE of these two species may have the same function.

In summary, the present study revealed the prevalence and clonal diversity of *S. argenteus* in northern Japan, together with presence of SE(-like) genes and other virulence factors. A novel staphylocoagulase genotype XV was identified for ST1223 clones. *S. argenteus* isolates harbored *ebpS*, *sdrE*, *selw*, *selx*, *sey*, *sel26*, and *sel27* that were phylogenetically distinct from those of *S. aureus*. This is the first comprehensive report on molecular epidemiological study of *S. argenteus* clinical isolates in Japan. Identification of emerging *S. argenteus* strains with three different clones with different profiles of virulence factors highlights the need for continuous surveillance of *S. argenteus* to understand its clinical significance.

## Figures and Tables

**Table 1:** Genotypes, virulence factors and drug resistance profile of *S. argenteus* isolates of Hokkaido, Japan (March to July 2019).

Isolate ID	Age/Sex	Specimen	City/Town ^a^	Inpatient/Outpatient	*coa* Genotype	ST	*spa* Type ^b^	*spa* Repeat Profile	Leukocidins, Haemolysins, Enterotoxins, TSST-1 ^c,d^	Adhesins, Modulators of Host Defense ^c,d^	Drug Resistance Genes ^e^	Antimicrobial Resistance Profile ^f^
SG01	81/F	sputum	A	Inpatient	XI-d	ST2250	t5078	299-31-25-17-17-16-16-16-16	*sec2, sey, selz, sel26, sel27*	*sdrC, sdrD*		
SG04	30/F	urine	B	Outpatient	XI-d	ST2250	NT	299-31-25-17-16-16-16	*sec3, sei, sel, sem, sey, selz, sel26, sel27, tst-1*	*sdrC, sak*		
SG05-1	46/F	ear discharge	A	Outpatient	XI-d	ST2250	NT	299-31-25-17-17-16	*sey, selz, sel26, sel27*	*sdrC, sdrD, sak*		
SG05-2	46/F	ear discharge	A	Outpatient	XI-d	ST2250	NT	299-31-25-17-17-16	*sey, selz, sel26, sel27*	*sdrC, sdrD, sak*		
SG09	14/M	nasal discharge	B	Outpatient	XI-d	ST2250	NT	N-17-17-16-16-16-16	*sey, selz, sel26, sel27*	*sdrC, sdrD*		
SG11	70/F	vaginal discharge	A	Outpatient	XI-d	ST2250	t17928	299-31-25-17-17-16-16-16	*sey, selz, sel26, sel27*	*sdrC, sdrD*		
SG16	N ^g^/F	stool	C	Outpatient	XI-d	ST2250	t5078	299-31-25-17-17-16-16-16-16	*selx, sey, sel26, sel27*	*sdrC, sdrD, sak*		
SG19	68/F	sputum	A	Inpatient	XI-d	ST2250	t5787	299-31-31-25-17-17-16-16-16-16	*sec3, sey, sel26, sel27, tst-1*	*sdrD*		
SG21	81/M	sputum	A	Outpatient	XI-d	ST2250	t7960	299-25-17-17-16-16-16-16	*sey*	*sdrC, sdrD*		
SG22	83/F	pharynx	A	Inpatient	XI-d	ST2250	t5078	299-31-25-17-17-16-16-16-16	*sey*	*sak, sdrC, sdrD*		
SG23	83/F	blood	A	Inpatient	XI-d	ST2250	t5078	299-31-25-17-17-16-16-16-16	*sey*	*sak, sdrC, sdrD*	*tet(L)*	TET, DOX
SG25	94/F	sputum	A	Inpatient	XI-d	ST2250	t17928	299-31-25-17-17-16-16-16	*sey*	*sdrC, sdrD*		
SG13	88/M	sputum	D	Inpatient	XI-d	ST3951 (ST2250 SLV)	t5078	299-31-25-17-17-16-16-16-16	*sey, selz, sel26, sel27*	*sdrD*		
SG02	75/F	urine	E	Outpatient	XIV	ST2198	NT	259-23-23-23-23-17-17-16	*selx*	*sdrC, sdrD*		
SG08	73/M	skin	F	Outpatient	XIV	ST2198	t7959	259-23-23-17-16	*selx, selz*	*sdrC*		
SG10	81/M	sputum	A	Inpatient	XIV	ST2198	t9385	259-366-23-17-17-17-23-23-368-17-16	*selx, selz*	*sdrC, sak*		
SG12	82/M	pharynx	G	Inpatient	XIV	ST2198	NT	259-17-16	*selx, selz*	*sdrC*		
SG14	75/M	skin	A	Outpatient	XIV	ST2198	NT	259-23-23-23-23-17-16	*selx*	*sdrC*		
SG20	74/M	pus	H	Outpatient	XIV	ST2198	NT	259-23-23-23-17-16	*selx*	*sdrC, sdrD*	*blaZ*	AMP
SG03	65/M	subdural abscess	D	Inpatient	XV	ST1223	t5142	259-25-17-17-16-16-16-17-16-16-16-16-16	*seg, sei, sem, sen, seo, selw, selz*	*sdrC, sdrD*		
SG06	73/M	nasal discharge	I	Outpatient	XV	ST1223	NT	259-25-16-16-16-16-16	*seg, sei, sem, sen, seo, selw, selz*	*sdrC, sdrD*		
SG07	17/M	stool	J	Outpatient	XV	ST1223	NT	259-25	*seg, sei, sem, sen, seo, selw, selz*	*sdrC*		
SG15	N/F	stool	A	Outpatient	XV	ST1223	t9791	259-25-17-17-16-16-17-16-16-16-16-16	*seg, sei, sem, sen, seo, selx, selw*	*sdrC, sdrD*		
SG17	32/M	stool	K	Outpatient	XV	ST1223	t7463	259-25-17-17-16-16-16-17-16-16-16-16	*seg, sei, sem, sen, seo, selw*	*sdrC, sdrD*		
SG18	N/F	vaginal discharge	A	Outpatient	XV	ST1223	t5142	259-25-17-17-16-16-16-17-16-16-16-16-16	*seg, sei, sem, sen, seo, selw*	*sdrC, sdrD*		

^a^ City/Town are shown by symbols A-K; ^b^ NT, non-typable (new *spa* allele); ^c^ The following genes were detected in all strains*: hla, hlb, hld, ebpS, eno, sdrE, fib, clfA, clfB, fnbA, fnbB, icaA;* ^d^ The following genes were not detected in any strain: *hlg, lukM, lukDE, lukS-PV-lukF-PV, sea, seb, sed, see, seh, sep, seq, ser, ses, set, seu, eta, etb, etd, icaD, cna, chp, bap, edn-A, edn-B, vWbp*; ^e^ The following genes were undetectable in any strain: *mecA*, *erm(A), erm(B), erm(C), msrA, tet(M), tet(K), aac(6′)-Ie-aph(2″)-Ia, aac(6′)-Im, ant(4′)-Ia, ant(9)-Ia, ant(9)-Ib, ant(3″)-Ia, aph(3′)-IIIa, aph(2″)-Ib, aph(2″)-Ic* and * aph(2″)-Id*; ^f^ Antimicrobials tested: ABK, Arbekacin; CFZ, Cefazolin; CLI, Clindamycin; CMZ, Cefmetazole; ERY, Erythormycin; FMX, Flomoxef: FOF, Fosfomycin; FOX, Cefoxitin; GEN, Gentamicin; IPM, Imipenem; LVX, Levofloxacin; LZD, Linezolid; MIN, Minocycline; TET, tetracycline; DOX, doxycycline; OXA, Oxacillin; SXT, Sulfamethoxazole-Trimethoprim; TEC, Teicoplanin; VAN, Vancomycin. ^g^ N, no information of patient’s age was available.

**Table 2:** Nucleotide sequence identities of staphylocoagulase genes (D1 and D2C regions) of *S. argenteus* isolates (SG03, SG06, SG17, SG18) to those of established *coa* genotypes of *S. arueus* and *S. argenteus* strains.

Strain	Species	GenBank Accession No.	*coa* Type ^a^	SG03		SG06		SG17		SG18	
				D1	D2C	D1	D2C	D1	D2C	D1	D2C
104	*S. aureus*	AB158549	Ia	68.8	69.1	68.8	68.8	68.8	69.1	68.8	69.2
213	*S. aureus*	X16457	IIa	66.5	70.6	66.5	70.3	66.5	70.6	66.5	70.7
SH682	*S. aureus*	EU105387	IIIa	66.0	71.4	66.0	71.1	66.0	71.4	66.0	71.5
Stp-28	*S. aureus*	AB158550	IVa	64.9	71.8	64.9	71.5	64.9	71.8	64.9	71.9
No.55	*S. aureus*	AB158551	Va	66.5	65.6	66.5	65.3	66.5	65.6	66.5	65.7
Stp-12	*S. aureus*	AB158552	VIa	88.3	76.8	88.3	76.5	88.3	76.8	88.3	76.9
St-1125	*S. aureus*	EU551129	VIIa	67.0	67.7	67.0	67.4	67.0	67.7	67.0	67.8
Ku	*S. aureus*	AB158553	VIIIa	69.3	68.8	69.3	68.5	69.3	68.8	69.3	68.9
17573	*S. aureus*	AB158554	IXa	66.1	70.6	66.1	70.3	66.1	70.6	66.1	70.7
19	*S. aureus*	AB158555	Xa	65.8	66.0	65.8	65.7	65.8	66.0	65.8	66.1
JCSC6075	*S. aureus*	AB436967	XIa	71.9	70.3	71.9	70.0	71.9	70.3	71.9	70.4
JCSC6669	*S. aureus*	AB436971	XIb	71.6	69.0	71.6	68.7	71.6	69.0	71.6	69.1
JCSC6990	*S. aureus*	AB742446	XIc	72.1	65.8	72.1	65.5	72.1	65.8	72.1	65.9
Tokyo 13310	*S. aureus*	LC122964	XI-variant	70.7	82.9	70.7	82.6	70.7	82.7	70.7	82.8
JCSC1469	*S. aureus*	AB436988	XIIa	67.7	78.7	67.7	78.4	67.7	78.7	67.7	78.8
mmr-v	*S. aureus*	KT599478	XIIIa	64.9	68.3	64.9	68.0	64.9	68.3	64.9	68.4
MSHR1132^T^	*S. argenteus*	FR821777	XII	67.7	78.7	67.7	78.4	67.7	78.7	67.7	78.8
S174	*S. argenteus*	MH484224	XId	70.7	82.9	70.7	82.6	70.7	82.7	70.7	82.8
RK308	*S. argenteus*	LSFQ01000033	XId	70.7	82.9	70.7	82.6	70.7	82.7	70.7	82.8
O-10	*S. argenteus*	FXVJ01000086	XId	70.7	82.9	70.7	82.6	70.7	82.7	70.7	82.8
XNO016	*S. argenteus*	CP025023	XId ^a^	70.7	82.9	70.7	82.6	70.7	82.7	70.7	82.8
XNO62	*S. argenteus*	CP023076	XId ^a^	70.7	82.9	70.7	82.6	70.7	82.7	70.7	82.8
BN75	*S. argenteus*	CP015758	XIV	67.0	82.8	67.0	82.5	67.0	82.6	67.0	82.7
S163	*S. argenteus*	MH484223	XIV	67.0	82.8	67.0	82.5	67.0	82.6	67.0	82.7
M260_MSHR	*S. argenteus*	CCEF01000001	XIV	67.0	82.8	67.0	82.5	67.0	82.5	67.0	82.6
SJTU F20124	*S. argenteus*	LWAN01000001	XV	100	100	100	99.7	100	99.8	100	99.9
D7903	*S. argenteus*	FXVL01000003	XV	100	100	100	99.7	100	99.8	100	99.9
M051_MSHR	*S. argenteus*	CCEN01000002	XV	100	100	100	99.7	100	99.8	100	99.9
SG03	*S. argenteus*	MN166536	XV	100	100	100	99.7	100	99.8	100	99.9
SG06	*S. argenteus*	MN166537	XV	100	99.7	100	100	100	99.4	100	99.5
SG17	*S. argenteus*	MN166540	XV	100	99.8	100	99.4	100	100	100	99.9
SG18	*S. argenteus*	MN166541	XV	100	99.9	100	99.5	100	99.9	100	100
NCTC13711	*S. argenteus*	UGZA01000002	NA	67.7	78.7	67.7	78.4	67.7	78.7	67.7	78.8
H1955	*S. argenteus*	FXWA01000108	NA	68.9	86.5	68.9	86.2	68.9	86.5	68.9	86.6
M5200	*S. argenteus*	FXVY01000005	NA	67.4	86.3	67.4	86.0	67.4	67.3	67.4	86.6

^a^*coa* genotype of these isolates were assigned to XId in our previous study [5]. *coa*-XV is a novel type identified in this study. NA, not assigned; presumptive *coa* type XVI was assigned in our previous study [5]; Nucleotide sequences of SG07 and SG15 were not included to this Table because they were 100% identical to that of SG03.

## Supplementary Materials

The supplementary materials are available online at https://www.mdpi.com/2076-2607/7/10/389/s1.
