# Supplementary material for: Molecular Epidemiological Characterization of Staphylococcus argenteus Clinical Isolates in Japan: Identification of Three Clones (ST1223, ST2198, and ST2550) and a Novel Staphylocoagulase Genotype XV
Source: Microorganisms. 2019 Sep 24;7(10):389. doi: 10.3390/microorganisms7100389 (PMC6843175; doi:10.3390/microorganisms7100389)
Supplement: Supplementary file 1 [file microorganisms-07-00389-s001.zip › Supplementary materials R1/Supple Fig-S2.docx]

**Figure S2** Alignment of elastin binding protein (EbpS) amino acid sequences from three *S. argenteus* isolates (SG03, ST1223; SG01, ST2250; SG20, ST2198) and three *S. aureus* strains (MW2, lineage I; LGA251, lineage Ia; COL, lineage II). Dot indicates identical amino acid, and dash denotes gap. LBR near N terminus represents ligand-binding region of EbpS. Three hydrophobic domains H1, H2 and H3 are shown in yellow.

**LBR**

SG03 --MSNNFKDDFEKNRQSIDTNSHQDHIEDVEKDQAEVEHQDTTEKTEQQFPPRNAQRRKR 58

SG01 --MSNNFKDDFEKNRQSIDTNSHQDHIEDVEKDQAEVEHQDTTEKTEQQFPPRNAQRRKR 58

SG20 MAMSNNFKDDFEKNRQSIDTNSHQDHIEDVEKDQAEVEHQDTTEKTEQQFPPRNAQRRKR 60

LGA251 --MSNNFKDDFEKNRQSIDTNSHQDHTEDVEKDQSELEHQDTTENTEQQFPPRNAQRRKR 58

COL --MSNNFKDDFEKNRQSIDTNSHQDHTEDVEKDQSELEHQDTIENTEQQFPPRNAQRRKR 58

MW2 --MSNNFKDDFEKNRQSIDTNSHQDHTEDVEKDQSELEHQDTIENTEQQFPPRNAQRRKR 58

************************ *******:*:***** *:***************

SG03 RRDLATNHNKQEHHESQVSEDKVQNEAGTLDDRQDESSLHSSLNQEASHHESKPHNDESN 118

SG01 RRDLATNHNKQEHQESQVSEDKVQNEAGTLDDRQDESSLHSSLNQEVSHHESKPHNDESN 118

SG20 RRDLATNHNKQEHQESQVSEDKVQNEAGTLDDRQDESSLHSSLNQEASHHESKPHNDESN 120

LGA251 RRDLATNHNKQHHDESQASDDNAHNEAVTLDDRQVESSAHSSVNQEPSHQDSTPQHEEEY 118

COL RRDLATNHNKQVHNESQTSEDNVQNEAGTIDDRQVESS-HSTESQEPSHQDSTPQHEEEY 117

MW2 RRDLATNHNKQVHNESQTSEDNVQNEAGTIDDRQVESS-HSTESQEPSHQDSTPQHEEEY 117

*********** *.***.*:*:.:*** *:**** *** **: .** **::*.*:::*.

SG03 YTKNAFAMDKSHPEPNEENVKHEEAQEERDTKENKSVANNVENEKVQQPKPHFSTDANKS 178

SG01 YTKNAFAMDKSHPEPNEENVKHEEAQEKRDTKENKSVANNVENEKVQQPKPHFSTDANKS 178

SG20 YTKNAFAMDKSHPEPNEENVKQEEAQEKRDTKENKSVANNVENEKVQQPKPHFSTDANKS 180

LGA251 YNKNAFAMDKSHPEPIEDNDKHETVKDAENNTEHSTVSDKSEAEQSQQPKPYFATGTNQA 178

COL YNKNAFAMDKSHPEPIEDNDKHDTIKNAENNTEHSTVSDKSEAEQSQQPKPYFTTGANQS 177

MW2 YNKNAFAMDKSHPEPIEDNDKHETIKEAENNTEHSTVSDKSEAEQSQQPKPYFATGANQA 177

*.************* *:* *:: :: .:..*:.:*::: * *: *****:*:*.:*::

**H1**

SG03 KESEENSNDVSLDSKNNDTKENHNGKKAAAIGAGKAGVAGAIAGASKA--NHHAKDQQNA 236

SG01 KESEENSNDVSLDSKNNDTKENHNGKKAAAIGAGTAGVAGAIGGASKA--NHHAKDQQNA 236

SG20 KESEESSNDVSLDSKNNDTKENHNGKKAAAIGAGTAGVAGAIGGASKA--NHHAKDQQNA 238

LGA251 NTSKDKHDDVTVKQDKDESKDHHSGKKGAAIGAGTAGVAGAAGAMAASKAKKHSNDAQNK 238

COL ETSKNEHDNDSVKQDQDEPKEHHNGKKAAAIGAGTAGVAGAAGAMAASKAKKHSNDAQNK 237

MW2 NTSKDKHDDVTVKQDKDESKDHHSGKKGAAIGAGTAGVAGAAGAMGVSKAKKHSNDAQNK 237

: *::. :: ::...::: *::*.***.******.****** .. . : ::*::* **

**H2**

SG03 QK-DNIEKSTESKEA-KKKEKDYNNKKGAAVGAGTAGAVGASAAAS-----KQQASAQTN 289

SG01 QK-DSIEKSTESKEA-KKKEKDYNNKKGAAVGAGTAGAVGASAAAS-----KQQASAQTN 289

SG20 QK-DSIEKSTESKEA-KKKEKDYNNKKGAAVGAGTAGAVGASAAAS-----KQQASAQTN 291

LGA251 SNSGKANNSTEDKVSQDKSKDHHNGKKGAAIGAGTAGLAGGAASKSASAASKPHASNNAS 298

COL SNSGKANNSTEDKASQDKSKDHHNGKKGAAIGAGTAGLAGGAASKSASAASKPHASNNAS 297

MW2 SNSDKSNNSTEDKVSQDKSKDHHNGKKGAAIGAGTAGLAGGAASKSASAASKPHASNNAS 297

.: .. ::***.* : .*.:..:*.*****:****** .*.:*: * * :** ::.

**H3**

SG03 HNHEHRDHHDKKKDHKKGGMTKVLLPLIAAVLIVGALAIFGGMALNNHNNGTKENKIANT 349

SG01 HNHEHRDHHDKKKDHKKGGMTKVLLPLIAAVLIVGALAIFGGMALNNHNNGTKENKIANT 349

SG20 HNHEHRDHHDKKKDHKKGGMTKVLLPLIAAVLIVGALAIFGGMALNNHNNGTKENKIANT 351

LGA251 QNHDEHDHHDRDKERKKGGMAKVLLPLIAAVLIIGALAIFGGMALNNHNNGTKENNIANT 358

COL QNHDEHDNHDRDKERKKGGMAKVLLPLIAAVLIIGALAIFGGMALNNHNNGTKENKIANT 357

MW2 QNHDEHDNHDRDKERKKGGMAKVLLPLIAAVLIIGALAIFGGMALNNHNNGTKENKIANT 357

:**:.:*:**:.*::*****:************:*********************:****

SG03 NKNKSDDDKDKATSTDSAKDKSKSEDNDKSKSDDKDKATTDESNNNQNTANQANNQTQNN 409

SG01 NKNKSDDNKDKATSTDSAKDKSKSEDNDKSKSDDKDKATTDESNNNQNTANQANNQTQNN 409

SG20 NKNKSDDNKDKATSTDSAKDKSKSEDNDKSKSDDKDKATTDESNNNQNTANQANNQTQNN 411

LGA251 NKNNADESKDKDTSKDASKDKSKSTDSDKSK-DDQDKATKDESDNDQNNANQANNQAQNN 417

COL NKNNADESKDKDTSKDASKDKSKSTDSDKSK-EDQDKATKDESDNDQNNANQANNQAQNN 416

MW2 NKNNADESKDKDTSKDASKDKSKSTDSDKSK-EDQDKATKDESDNDQNNANQANNQAQNN 416

***::*:.*** **.*::****** *.**** :*:****.***:*:**.*******:***

SG03 QNQQQANQNQ--QQQRQGGGQRHTVSGQENLYRIAIQYYGSGSPENVEKIRRANGLSGNN 467

SG01 QNQQQANQNQ--QQQRQGGGQRHTVSGQENLYRIAIQYYGSGSPENVEKIRRANGLSGNN 467

SG20 QNQQQANQNQ--QQQRQGGGQRHTVSGQENLYRIAIQYYGSGSPENVEKIRRANGLSGNN 469

LGA251 QNQQQANQNQQQQQQRQGGGQRHTVNGQENLYRIAIQYYGSGSPENVEKIRRANGLSGNN 477

COL QNQQQANQNQQQQQQRQGGGQRHTVNGQENLYRIAIQYYGSGSPENVEKIRRANGLSGNN 476

MW2 QNQQQANQNQQQQQQRQGGGQRHTVNGQENLYRIAIQYYGSGSPENVEKIRRANGLSGNN 476

********** *************.**********************************

SG03 IINGQQIIIP 477

SG01 IRNGQQIIIP 477

SG20 IRNGQQIIIP 479

LGA251 IRNGQQIVIP 487

COL IRNGQQIVIP 486

MW2 IRNGQQIVIP 486

* *****:**
