# Supplementary material for: Molecular Epidemiological Characterization of Staphylococcus argenteus Clinical Isolates in Japan: Identification of Three Clones (ST1223, ST2198, and ST2550) and a Novel Staphylocoagulase Genotype XV
Source: Microorganisms. 2019 Sep 24;7(10):389. doi: 10.3390/microorganisms7100389 (PMC6843175; doi:10.3390/microorganisms7100389)
Supplement: Supplementary file 1 [file microorganisms-07-00389-s001.zip › Supplementary materials R1/Supple Fig-S3.docx]

**Figure S3** Alignment of SdrE amino acid sequences from eleven *S. argenteus* strains (filled circle, isolates analyzed in the present study) and two *S. aureus* strains shown with open circle. Asterisk below the alignment indicates identical amino acid, while dash denotes gap. Lineages I and II identified in the phylogenetic tree (Fig.S8) are shown on the right of the first row of the alignment. Signal peptide, Region A, B repeats (B1, B2, B3), SD repeat region, W and M regions (short wall-spanning region, membrane-spanning region, respectively) are indicated on the alignment. TYTFTDYVD motif and LPXTG motif are shown in yellow and purple, respectively. CnaB domain located in the B3 region is boxed, and amino acids different from those of strain MSHR1132 are shown in green. Amino acid numbers are shown on the right. Because sequence of S. argenteus strain BN75 available in GenBank had 100bp gap, amino acid sequence is not complete.

**Signal peptide ←▐→ Region A**

●SG02 MINRDNKKAITKKGMISNRLNKFSIRKYTVGTASILVGTTLIFGLGNQEAKAAENTSTEN 60 **lineage II**

MSHR1132_FR821777 MINRDNKKAITKKGMISNRLNKFSIRKYTVGTASILVGTTLIFGLGNQEAKAAENTSTEN 60 **lineage II**

●SG14 MINRDNKKAITKKGMISNRLNKFSIRKYTVGTASILVGTTLIFGLGNQEAKAAENTSTEN 60 **lineage II**

●SG12 MINRDNKKAITKKGMISNRLNKFSIRKYTVGTASILVGTTLIFGLGNQEAKAAENTSTEN 60 **lineage II** BN75(CP015758) MINRDNKKAITKKGMISNRLNKFSIRKYTVGTASILVGTTLIFGLGNQEAKAAENTSTEN 60 **lineage II**

○H-EMRSA-15(CP007659) MINRDNKKAITKKGMISNRLNKFSIRKYTVGTASILVGTTLIFGLGNQEAKAAENTSTEN 60 **lineage I**

●SG06 MINRDNKKAITKKGMISNRLNKFSIRKYTVGTASILVGTTLIFGLGNQEAKAAENTNTEN 60 **lineage I**

○DAR4145(CP010526) MINRDNKKAITKKGMISNRLNKFSIRKYTVGTASILVGTTLIFGLGNQEAKAAENTSTEN 60 **lineage I**

●SG03 MINRDNKKAITKKGMISNRLNKFSIRKYTVGTASILVGTTLIFGLGNQEAKAAENTNTEN 60 **lineage I**

●SG07 MINRDNKKAITKKGMISNRLNKFSIRKYTVGTASILVGTTLIFGLGNQEAKAAENTSTEN 60 **lineage I**

●SG13 --------------MISNRLNKFSIRKYTVGTASILVGTTLIFGLGNQEAKAAENTSTEN 46 **lineage I**

●SG11 MINRDNKKAITKKGMISNRLNKFSIRKYTVGTASILVGTTLIFGLGNQEAKAAENTSTEN 60 **lineage I**

●SG04 MINRDNKKAITKKGMISNRLNKFSIRKYTVGTASILVGTTLIFGLGNQEAKAAENTSTEN 60 **lineage I**

58113 --------------MISNRLNKFSIRKYTVGTASILVGTTLIFGLGNQEAKAAENTSTEN 46 **lineage I**

******************************************.***

SG02 AKQDEASTSADKEVMSNTENNSTTEKNTTDSIKEETNTDSQTEVKEEPKTSTTQQQQNNT 120

MSHR1132_FR821777 AKQDEASTSADKEVMSNTENNSTTEKNTTDSIKEETNTDSQTEVKEEPKTSTTQQQQNNT 120

SG14 AKQDEASTSADKEVMSNTENNSTTEKNTTDSIKEETNTDSQTEVKEEPKTSTTQQQQNNT 120

SG12 AKQDEASTSADKEVMSNTENNSTTEKNTTDSIKEETNTDSQTEVKEEPKTSTTQQQQNNT 120

BN75(CP015758) AKQDEASTSADKEVMSNTENNSTTEKNTTDSIKEETNTDSQTEVKEEPKTSTTQQQQNNT 120

H-EMRSA-15(CP007659) AKQDDATTSDNKEVVSEAENNSTTENDSTNPIKKETNTDSQPEAKEESTTSSTQQQQNNV 120

SG06 AKQDEASTIADKEVMSNTENNSTTEKNTTDSIKEETNTDSQIEVKEEPKTSTTQQQQNNT 120

DAR4145(CP010526) AKQDDATTSDNKEVVSETENNSTTENNSTNPIKKETNTDSQPEAKKESTSSSTQKQQNNV 120

SG03 AKQDEASTIADKEVMSNTENNSTTEKNTTDSIKEETNTDSQIEVKEEPKTSTTQQQQNNT 120

SG07 AKQDEASTSADKEVMSNTENNSTTEKNTTDSIKEETNTDSQIEVKEEPKTSTTQQQQNNT 120

SG13 AKQDEASTSADKEVMSNTENNSTTEKNATDSIKEETNTDSQTEVKEEPKTSTTQQQQNNT 106

SG11 AKQDEASTSADKEVMSNTENNSTTEKNATDSIKEETNTDSQTEVKEEPKTSTTQQQQNNT 120

SG04 AKQDEASTSADKEVMSNTENNSTTEKNATDSIKEETNTDSQTEVKEEPKTSTTQQQQNNT 120

58113 AKQDEASTSADKEVMSNTENNSTTEKNATDSIKEETNTDSQTEVKEEPKTSTTQQQQNNT 106

****:*:* :***:*::*******:::*: **:******* *.*:* .:*:**:****.

SG02 TTSTETKPQINENNNVKPSTDKTATEDTSVILEEKTAPNNKNNDVTTNQSTSETQPTQQT 180

MSHR1132_FR821777 TTSTETKPQTNENNNVKPSTDKTATEDTSVILEEKTAPNNKNNDVTTNQSTSETQPTQQT 180

SG14 TTSTETKPQINENNNVKPSTDKTATEDTSVILEEKTAPNNKNNDVTTNQSTSETQPTQQT 180

SG12 TTSTETKPQINENNNVKPSTDKTATEDTSVILEEKTAPNNKNNDVTTNQSTSETQPTQQT 180

BN75(CP015758) TTSTETKPQINENNNVKPSTDKTATEDTSVILEEKTAPNNKNNDVTTNQSTSETQPTQQT 180

H-EMRSA-15(CP007659) TATTETKPQNIEKENVKPSTDKTATEDTSVILEEKKAPNNTNNDVTTKPSTSEIQKKPTT 180

SG06 TTSTETKPQTNENNNVKPSTDKTATEDTSVILEEKTAPNNKNNDVTTNQSTSETQPTQQT 180

DAR4145(CP010526) TATTETKPQNIEKENVKPSTDKTATEDTSVILEEKKAPNNTNNDVTTKPSTSEIQTKPTT 180

SG03 TTSTETKPQTNENNNVKPSTDKTATEDTSVILEEKTAPNNKNNDVTTNQSTSETQPTQQT 180

SG07 TTSTETKPQTNENNNVKPSTDKTATEDTSVILEEKTAPNNKNNDVTTNQSTSETQPTQQT 180

SG13 TTSTETKPQTNENNNVKPSTDKTATEDTSVILEEKTAPNNKNNDVTTNQSTSETQPTQQT 166

SG11 TTSTETKPQTNENNNVKPSTDKTATEDTSVILEEKTAPNNKNNDVTTNQSTSETQPTQQT 180

SG04 TTSTETKPQTNENNNVKPSTDKTATEDTSVILEEKTAPNNKNNDVTTNQSTSETQPTQQT 180

58113 TTSTETKPQTNENNNVKPSTDKTATEDTSVILEEKTAPNNKNNDVTTNQSTSETQPTQQT 166

*::****** *::*********************.****.******: **** * . *

SG02 PQEPTNNETSQPQSTPSKVDNQVTDATNPKQTVNVSKEDLKNDPEKLKELVRSENNTDHS 240

MSHR1132_FR821777 PQEPTNNETSQPQSTPSKVDNQVTDATNPKQTVNVSKEDLKNDPEKLKELVRSENNTDHS 240

SG14 PQEPTNNETSQPQSTPSKVDNQVTDATNPKQTVNVSKEDLKNDPEKLKELVRSENNTDHS 240

SG12 PQEPTNNETSQPQSTPSKVDNQVTDATNPKQTVNVSKEDLKNDPEKLKELVRSENNTDHS 240

BN75(CP015758) PQEPTNNETSQPQSTPSKVDNQVTDATNPKQTVNVSKEDLKNDPEKLKELVRSENNTDHS 240

H-EMRSA-15(CP007659) PQESTNIENSQPQPTPSKVDNQVTDATNPKEPVNVSKEELKNNPEKLKELVRNDSNTDHS 240

SG06 PQEPTNNETSQPQSTPSNVDNQVTDATNPKQTVNVSKEDLKNDPEKLKELVRSENNTDHS 240

DAR4145(CP010526) PQESTNIENSQSQPTPSKVDNQVTDATNPKQTVNVSKEDLKNDPEKLKELVRSENNTNHS 240

SG03 PQEPTNNETSQPQSTPSNVDNQVTDATNPKQTVNVSKEDLKNDPEKLKELVRSENNTDHS 240

SG07 PQEPTNNETSQPQSTPSNVDNQVTDATNPKQTVNVSKEDLKNDPEKLKELVRSENNTDHS 240

SG13 PQEPTNNETSQPQSTPSKVDNQVTDATNPKQTVNVSKEDLKNDPEKLKELVRSENNTDHS 226

SG11 PQEPTNNETSQPQSTPSKVDNQVTDATNPKQTVNVSKEDLKNDPEKLKELVRSENNTDHS 240

SG04 PQEPTNNETSQPQSTPSKVDNQVTDATNPKQTVNVSKEDLKNDPEKLKELVRSENNTDHS 240

58113 PQEPTNNETSQPQSTPSKVDNQVTDATNPKQTVNVSKEDLKNDPEKLKELVRSENNTDHS 226

*** ** *.** * ***:************: ******:***:*********.:.**:**

SG02 TKPVATAPTSVAPKRLNAKMRFAVAQPAAVASNNVNDLITVTKQTITEGIKDDGVIQAHD 300

MSHR1132_FR821777 TKPVATAPTSVAPKRLNAKMRFAVAQPAAVASNNVNDLITVTKQTITEGIKDDGVIQAHD 300

SG14 TKPVATAPTSVAPKRLNAKMRFAVAQPAAVASNNVNDLITVTKQTITEGIKDDGVIQAHD 300

SG12 TKPVATAPTSVAPKRLNAKMRFAVAQPAAVASNNVNDLITVTKQTITEGIKDDGVIQAHD 300

BN75(CP015758) TKPVATAPTSVAPKRLNAKMRFAVAQPAAVASNNVNDLITVTKQTITEGIKDDGVIQAHD 300

H-EMRSA-15(CP007659) TKPVATAPTSVAPKRVNAKMRFAVAQPAAVASNNVNDLIKVTKQTIKVGDGKDNVAAAHD 300

SG06 TKPVATAPTSVAPKRLNAKMRFAVAQPAAVASNNVNDLITVTKQTIKVGDGTDNVAAAHD 300

DAR4145(CP010526) TKPVATAPTSVAPKRLNAKMRFAVAQPAAVASNNVNDLITVTKQTIKVGDGKDNVAAAHD 300

SG03 TKPVATAPTSVAPKRLNAKMRFAVAQPAAVASNNVNDLITVTKQTIKVGDGTDNVAAAHD 300

SG07 TKPVATAPTSVAPKRLNAKMRFAVAQPAAVASNNVNDLITVTKQTIKVGDGTDNVAAAHD 300

SG13 TKPVATAPTSVAPKRLNAKMRFAVAQPAAVASNNVNDLITVTKQTIKVGDGTDNVAAAHD 286

SG11 TKPVATAPTSVAPKRLNAKMRFAVAQPAAVASNNVNDLITVTKQTIKVGDGTDNVAAAHD 300

SG04 TKPVATAPTSVAPKRLNAKMRFAVAQPAAVASNNVNDLITVTKQTIKVGDGTDNVAAAHD 300

58113 TKPVATAPTSVAPKRLNAKMRFAVAQPAAVASNNVNDLITVTKQTIKVGDGTDNVAAAHD 286

***************:***********************.******. * *.* ***

SG02 GEHIIYTSDFKIDNAVKAGDTMTVKYDKHTIPSDITDDFTPVDITDPSGEVIAKGTFDLN 360

MSHR1132_FR821777 GEHIIYTSDFKIDNAVKAGDTMTVKYDKHTIPSDITDDFTPVDITDPSGEVIAKGTFDLN 360

SG14 GEHIIYTSDFKIDNAVKAGDTMTVKYDKHTIPSDITDDFTPVDITDPSGEVIAKGTFDLN 360

SG12 GEHIIYTSDFKIDNAVKAGDTMTVKYDKHTIPSDITDDFTPVDITDPSGEVIAKGTFDLN 360

BN75(CP015758) GEHIIYTSDFKIDNAVKAGDTMTVKYDKHTIPSDITDDFTPVDITDPSGEVIAKGTFDLN 360

H-EMRSA-15(CP007659) GKDIEYDTEFTIDNKVKKGDTMTINYDKNVIPSDLTDKNDPIDITDPSGEVIAKGTFDKA 360

SG06 GKDIEYDTEFTIDNKVKKGDTMTINYDKNVIPSDLTDKNDPIDITDPSGEVIAKGTFDKA 360

DAR4145(CP010526) GKDIEYDTEFTIDNKVKKGDTMTINYDKNVIPSDLTDKNDPIDITDPSGEVIAKGTFDKA 360

SG03 GKDIEYDTEFTIDNKVKKGDTMTINYDKNVIPSDLTDKNDPIDITDPSGEVIAKGTFDKA 360

SG07 GKDIEYDTEFTIDNKVKKGDTMTINYDKNVIPSDLTDKNDPIDITDPSGEVIAKGTFDKA 360

SG13 GKDIEYDTEFTIDNKVKKGDTMTINYDKNVIPSDLTDKNDPIDITDPSGEVIAKGTFDKA 346

SG11 GKDIEYDTEFTIDNKVKKGDTMTINYDKNVIPSDLTDKNDPIDITDPSGEVIAKGTFDKA 360

SG04 GKDIEYDTEFTIDNKVKKGDTMTINYDKNVIPSDLTDKNDPIDITDPSGEVIAKGTFDKA 360

58113 GKDIEYDTEFTIDNKVKKGDTMTINYDKNVIPSDLTDKNDPIDITDPSGEVIAKGTFDKA 346

*:.* * ::*.*** ** *****::***:.****:**. *:****************

**TYTFTDYVD motif**

SG02 TKTITYKFTDYVDRYENVNAKLELNSYIDKKEVPNETNLNLTFATADKETSKNVKVEYQK 420

MSHR1132_FR821777 TKTITYKFTDYVDRYENVNAKLELNSYIDKKEVPNETNLNLTFATADKETSKNVKVEYQK 420

SG14 TKTITYKFTDYVDRYENVNAKLELNSYIDKKEVPNETNLNLTFATADKETSKNVKVEYQK 420

SG12 TKTITYKFTDYVDRYENVNAKLELNSYIDKKEVPNETNLNLTFATADKETSKNVKVEYQK 420

BN75(CP015758) TKTITYKFTDYVDRYENVNAKLELNSYIDKKEVPNETNLNLTFATADKETSKNVKVEYQK 420

H-EMRSA-15(CP007659) TKQITYTFTDYVDKYEDIKSRLTLYSYIDKKTVPNETSLNLTFATAGKETSQNVTVDYQD 420

SG06 TKQITYTFTDYVDKYEDIKSRLTLYSYIDKQVVPNETSLNLTFATAGKETSKNVTVDYQD 420

DAR4145(CP010526) TKQITYTFTDYVDKYEDIKARLTLYSYIDKQAVPNETSLNLTFATAGKETSQNVSVDYQD 420

SG03 TKQITYTFTDYVDKYEDIKSRLTLYSYIDKQVVPNETSLNLTFATAGKETSKNVTVDYQD 420

SG07 TKQITYTFTDYVDKYEDIKSRLTLYSYIDKQVVPNETSLNLTFATAGKETSKNVTVDYQD 420

SG13 TKQITYTFTDYVDKYEDIKSRLTLYSYIDKQVVPNETSLNLTFATAGKETSQNVTVDYQD 406

SG11 TKQITYTFTDYVDKYEDIKSRLTLYSYIDKQVVPNETSLNLTFATAGKETSQNVTVDYQD 420

SG04 TKQITYTFTDYVDKYEDIKSRLTLYSYIDKQVVPNETSLNLTFATAGKETSQNVTVDYQD 420

58113 TKQITYTFTDYVDKYEDIKSRLTLYSYIDKQVVPNETSLNLTFATAGKETSQNVTVDYQD 406

** ***.******:**:::::* * *****: *****.********.****:**.*:**.

SG02 PIVKDESNIQSIFSHLDTTKHEVEQTIYVNPLKLNAKNTNVTIKSGGVADNGDYYTGDGS 480

MSHR1132_FR821777 PIVKDESNIQSIFSHLDTTKHEVEQTIYVNPLKLNAKNTNVTIKSGGVADNGDYYTGDGS 480

SG14 PIVKDESNIQSIFSHLDTTKHEVEQTIYVNPLKLNAKNTNVTIKSGGVADNGDYYTGDGS 480

SG12 PIVKDESNIQSIFSHLDTTKHEVEQTIYVNPLKLNAKNTNVTIKSGGVADNGDYYTGDGS 480

BN75(CP015758) PIVKDESNIQSIFSHLDTTKHEVEQTIYVNPLKLNAKNTNVTIKSGGVADNGDYYTGDGS 480

H-EMRSA-15(CP007659) PMVHGDSNIQSIFTKLDEDKQTIEQQIYVNPLKKSATNTKVDIAGSQVDDYGNIKLGNGS 480

SG06 PMVHGDSNIQSIFTKLDEDKQTIEQQIYVNPLKKTATNTKVDIAGSQVDDYGNIKSGNGS 480

DAR4145(CP010526) PMVHGDSNIQSIFTKLDENKQTIEQQIYVNPLKKTATNTKVDIAGSQVDDYGNIKLGNGS 480

SG03 PMVHGDSNIQSIFTKLDEDKQTIEQQIYVNPLKKTATNTKVDIAGSQVDDYGNIKSGNGS 480

SG07 PMVHGDSNIQSIFTKLDEDKQTIEQQIYVNPLKKTATNTKVDIAGSQVDDYGNIKSGNGS 480

SG13 PMVHGDSNIQSIFTKLDEDKQTIEQQIYVNPLKKTATNTKVDIAGSQVDDYGNIKLGNGS 466

SG11 PMVHGDSNIQSIFTKLDEDKQTIEQQIYVNPLKKTATNTKVDIAGSQVDDYGNIKLGNGS 480

SG04 PMVHGDSNIQSIFTKLDEDKQTIEQQIYVNPLKKTATNTKVDIAGSQVDDYGNIKLGNGS 480

58113 PMVHGDSNIQSIFTKLDEDKQTIEQQIYVNPLKKTATNTKVDIAGSQVDDYGNIKLGNGS 466

*:*:.:*******::** *: :** ******* .*.**:* * .. * * *: *:**

SG02 TIIDSNTEIKVYKVASGQQLPQSNKIYDYSQYEDVTNSVTINKNYGANMANINFGDIDSA 540

MSHR1132_FR821777 TIIDSNTEIKVYKVASGQQLPQSNKIYDYSQYEDVTNSVTINKNYGANMANINFGDIDSA 540

SG14 TIIDSNTEIKVYKVASGQQLPQSNKIYDYSQYEDVTNSVTINKNYGANMANINFGDIDSA 540

SG12 TIIDSNTEIKVYKVASGQQLPQSNKIYDYSQYEDVTNSVTINKNYGANMANINFGDIDSA 540

BN75(CP015758) TIIDSNTEIKVYKVASGQQLPQSNKIYDYSQYEDVTNSVTINKNYGANMANINFGDIDSA 540

H-EMRSA-15(CP007659) TIIDQNTEIKVYKVNSDQQLPQSNRIYDFSQYEDVTSQFDNKKSFSNNVATLDFGDINSA 540

SG06 TIIDQNTEIKVYKVNPNQQLPQSNRIYDFSQYEDVTSQFDNKKSFSNNVATLDFGDINSA 540

DAR4145(CP010526) TIIDQNTEIKVYKVNPNQQLPQSNRIYDFSQYEDVTSQFDNKKSFSNNVATLDFGDINSA 540

SG03 TIIDQNTEIKVYKVNPNQQLPQSNRIYDFSQYEDVTSQFDNKKSFSNNVATLDFGDINSA 540

SG07 TIIDQNTEIKVYKVNPNQQLPQSNRIYDFSQYEDVTSQFDNKKSFSNNVATLDFGDINSA 540

SG13 TIIDQNTEIKVYKVNPNQQLPQSNRIYDFSQYEDVTSQFDNKKSFSNNVATLDFGDINSA 526

SG11 TIIDQNTEIKVYKVNPNQQLPQSNRIYDFSQYEDVTSQFDNKKSFSNNVATLDFGDINSA 540

SG04 TIIDQNTEIKVYKVNPNQQLPQSNRIYDFSQYEDVTSQFDNKKSFSNNVATLDFGDINSA 540

58113 TIIDQNTEIKVYKVNPNQQLPQSNRIYDFSQYEDVTSQFDNKKSFSNNVATLDFGDINSA 526

****.********* .*******:***:*******... :*.:. *:*.::****:**

SG02 YIVKVVSKYTPGAEDDLAVQQGVRMTTTNKYNYSSYAGYTNTILSTTDSGGGDGTVKPEE 600

MSHR1132_FR821777 YIVKVVSKYTPGAEDDLAVQQGVRMTTTNKYNYSSYAGYTNTILSTTDSGGGDGTVKPEE 600

SG14 YIVKVVSKYTPGAEDDLAVQQGVRMTTTNKYNYSSYAGYTNTILSTTDSGGGDGTVKPEE 600

SG12 YIVKVVSKYTPGAEDDLAVQQGVRMTTTNKYNYSSYAGYTNTILSTTDSGGGDGTVKPEE 600

BN75(CP015758) YIVKVVSKYTPGAEDDLAVQQGVRMTTTNKYNYSSYAGYTNTILSTTDSGGGDGTVKPEE 600

H-EMRSA-15(CP007659) YIIKVVSKYTPTSDGELDIAQGTSMRTTDKYGYYNYAGYSNFIVTSNDSGGGDGTVKPEE 600

SG06 YIIKVVSKYTPTSDGELDIAQGTSMRTTDKYGYYNYAGYSNFIVTSNDSGGGDGTVKPEE 600

DAR4145(CP010526) YIIKVVSKYTPTSDGELDIAQGTSMRTTDKYGYYNYAGYSNFIVTSNDSGGGDGTVKPEE 600

SG03 YIIKVVSKYTPTSDGELDIAQGTSMRTTDKYGYYNYAGYSNFIVTSNDSGGGDGTVKPEE 600

SG07 YIIKVVSKYTPTSDGELDIAQGTSMRTTDKYGYYNYAGYSNFIVTSNDSGGGDGTVKPEE 600

SG13 YIIKVVSKYTPTSDGELDIAQGTSMRTTDKYGYYNYAGYSNFIVTSNDSGGGDGTVKPEE 586

SG11 YIIKVVSKYTPTSDGELDIAQGTSMRTTDKYGYYNYAGYSNFIVTSNDSGGGDGTVKPEE 600

SG04 YIIKVVSKYTPTSDGELDIAQGTSMRTTDKYGYYNYAGYSNFIVTSNDSGGGDGTVKPEE 600

58113 YIIKVVSKYTPTSDGELDIAQGTSMRTTDKYGYYNYAGYSNFIVTSNDSGGGDGTVKPEE 586

**:******** ::.:* : **. * **:**.* .****:* *:::.*************

▐→B1

SG02 KLYKIGDYVWEDVDKDGVQGTDSKEKPMANVLVTLTYPDGTTKSVRTDANGHYEFGGLKD 660

MSHR1132_FR821777 KLYKIGDYVWEDVDKDGVQGTDSKEKPMANVLVTLTYPDGTTKSVRTDANGHYEFGGLKD 660

SG14 KLYKIGDYVWEDVDKDGVQGTDSKEKPMANVLVTLTYPDGTTKSVRTDANGHYEFGGLKD 660

SG12 KLYKIGDYVWEDVDKDGVQGTDSKEKPMANVLVTLTYPDGTTKSVRTDANGHYEFGGLKD 660

BN75(CP015758) KLYKIGDYVWEDVDKDGVQGTDSKEKPMANVLVTLTYPDGTTKSVRTDANGHYEFGGLKD 660

H-EMRSA-15(CP007659) KLYKIGDYVWEDVDKDGVQGTDSKEKPMANVLVTLTYPDGTTKSVRTDANGHYEFGGLKD 660

SG06 KLYKIGDYVWEDVDKDGVQGTDSKEKPMANVLVTLTYPDGTTKSVRTDANGHYEFGGLKD 660

DAR4145(CP010526) KLYKIGDYVWEDVDKDGVQGTDSKEKPMANVLVTLTYPDGTTKSVRTDANGHYEFGGLKD 660

SG03 KLYKIGDYVWEDVDKDGVQGTDSKEKPMANVLVTLTYPDGTTKSVRTDANGHYEFGGLKD 660

SG07 KLYKIGDYVWEDVDKDGVQGTDSKEKPMANVLVTLTYPDGTTKSVRTDAKGHYEFGGLKD 660

SG13 KLYKIGDYVWEDVDKDGVQGTDSKEKPMANVLVTLTYPDGTTKSVRTDAKGHYEFGGLKD 646

SG11 KLYKIGDYVWEDVDKDGVQGTDSKEKPMANVLVTLTYPDGTTKSVRTDAKGHYEFGGLKD 660

SG04 KLYKIGDYVWEDVDKDGVQGTDSKEKPMANVLVTLTYPDGTTKSVRTDAKGHYEFGGLKD 660

58113 KLYKIGDYVWEDVDKDGVQGTDSKEKPMANVLVTLTYPDGTTKSVRTDAKGHYEFGGLKD 646

*************************************************:**********

▐→B2

SG02 GETYTVKFETPAGYLPTKENGTTDGEKDSNGSSVTVKINGKDDMSLDSGFYKEPKYNLGD 720

MSHR1132_FR821777 GETYTVKFETPAGYLPTKENGTTDGEKDSNGSSVTVKINGKDDMSLDSGFYKEPKYNLGD 720

SG14 GETYTVKFETPAGYLPTKENGTTDGEKDSNGSSVTVKINGKDDMSLDSGFYKEPKYNLGD 720

SG12 GETYTVKFETPAGYLPTKENGTTDGEKDSNGSSVTVKINGKDDMSLDSGFYKEPKYNLGD 720

BN75(CP015758) GETYTVKFETPAGYLPTKENGTTDGEKDSNGSSVTVKINGKDDMSLDSGFYKEPKYNLGD 720

H-EMRSA-15(CP007659) GETYTVKFETPAGYLPTKENGTTDGEKDSNGSSVTVKINGKDDMSLDTGFYKEPKYNLGD 720

SG06 GETYTVKFETPAGYLPTKENGTTDGEKDSNGSSVTVKINGKDDMSLDSGFYKEPKYNLGD 720

DAR4145(CP010526) GETYTVKFETPAGYLPTKVNGTTDGEKDSNGSSVTVKINGKDDMSLDTGFYKEPKYNLGD 720

SG03 GETYTVKFETPAGYLPTKENGTTDGEKDSNGSSVTVKINGKDDMSLDSGFYKEPKYNLGD 720

SG07 GETYTVKFETPAGYLPTKENGTTDGEKDSNGSSVTVKINGKDDMSLDSGFYKEPKYNLGD 720

SG13 GETYTVKFETPAGYLPTKENGTTDGEKDSNGSSVTVKINGKDDMSLDSGFYKEPKYNLGD 706

SG11 GETYTVKFETPAGYLPTKENGTTDGEKDSNGSSVTVKINGKDDMSLDSGFYKEPKYNLGD 720

SG04 GETYTVKFETPAGYLPTKENGTTDGEKDSNGSSVTVKINGKDDMSLDSGFYKEPKYNLGD 720

58113 GETYTVKFETPAGYLPTKENGTTDGEKDSNGSSVTVKINGKDDMSLDSGFYKEPKYNLGD 706

****************** ****************************:************

SG02 YVWEDTNKDGIQDANEPGIKDVKVTLKDSTGKIIGTTTTDASGKYKFTDLDNGNYTVEFE 780

MSHR1132_FR821777 YVWEDTNKDGIQDANEPGIKDVKVTLKDSTGKIIGTTTTDASGKYKFTDLDNGNYTVEFE 780

SG14 YVWEDTNKDGIQDANEPGIKDVKVTLKDSTGKIIGTTTTDASGKYKFTDLDNGNYTVEFE 780

SG12 YVWEDTNKDGIQDANEPGIKDVKVTLKDSTGKIIGTTTTDASGKYKFTDLDNGNYTVEFE 780

BN75(CP015758) YVWEDTNKDGIQDANEPGIKDVKVTLKDSTGKIIGTTTTDASGKYKFTDLDNGNYTVEFE 780

H-EMRSA-15(CP007659) YVWEDTNKDGIQDANEPGIKDVKVTLKDSTGKVIGTTTTDASGKYKFTDLDNGNYTVEFE 780

SG06 YVWEDTNKDGIQDANEPGIKDVKVTLKDSTGKIIGTTTTDASGKYKFADLDNGNYTVEFE 780

DAR4145(CP010526) YVWEDTNKDGIQDANEPGIKDVKVTLKDSTGKVIGTTTTDASGKYKFTDLDNGNYTVEFE 780

SG03 YVWEDTNKDGIQDANEPGIKDVKVTLKDSTGKIIGTTTTDASGKYKFTDLDNGNYTVEFE 780

SG07 YVWEDTNKDGIQDANEPGIKDVKVTLKDSTGKIIGTTTTDASGKYKFTDLDNGNYTVEFE 780

SG13 YVWEDTNKDGIQDANEPGIKDVKVTLKDSTGKIIGTTTTDASGKYKFTDLDNGNYTVEFE 766

SG11 YVWEDTNKDGIQDANEPGIKDVKVTLKDSTGKIIGTTTTDASGKYKFTDLDNGNYTVEFE 780

SG04 YVWEDTNKDGIQDANEPGIKDVKVTLKDSTGKIIGTTTTDASGKYKFTDLDNGNYTVEFE 780

58113 YVWEDTNKDGIQDANEPGIKDVKVTLKDSTGKIIGTTTTDASGKYKFTDLDNGNYTVEFE 766

********************************:**************:************

　　　　　　　　　　　　　　　　　　　　　　　　　　　　　　　　　　　▐→B3

SG02 TPAGYTPTLKNTTAEDKDSNGLTTTGVIKDADNWTLDSGFYKTPKYSLGDYVWYDSNKDG 840

MSHR1132_FR821777 TPAGYTPTLKNTTAEDKDSNGLTTTGVIKDADNWTLDSGFYKTPKYSLGDYVWYDSNKDG 840

SG14 TPAGYTPTLKNTTAEDKDSNGLTTTGVIKDADNWTLDSGFYKTPKYSLGDYVWYDSNKDG 840

SG12 TPAGYTPTLKNTTAEDKDSNGLTTTGVIKDADNWTLDSGFYKTPKYSLGDYVWYDSNKDG 840

BN75(CP015758) TPAGYTPTLKNTTAEDKDSNGLTTTGVIKDADNWTLDSGFYKTPKYSLGDYVWYDSNKDG 840

H-EMRSA-15(CP007659) TPAGYTPTVKNTTAEDKDSNGLTTTGVIKDADNMTLDSGFYKTPKYSLGDYVWYDSNKDG 840

SG06 TPAGYTPTLKNTTAEDKDSNGLTTAGVIKDADDWTLDSGFYKKPKYNLGDYVWYDSNQDG 840

DAR4145(CP010526) TPAGYTPTVKNTTADDKDSNGLTTTGVIKDADNMTLDSGFYKTPKYSLGDYVWYDSNKDG 840

SG03 TPAGYTPTLKNTTAEDKDSNGLTTTGVIKDADNWTLDSGFYKTPKYSLGDYVWYDSNKDG 840

SG07 TPAGYTPTLKNTTAEDKDSNGLTTTGVIKDADNWTLDSGFYKTPKYSLGDYVWYDSNKDG 840

SG13 TPAGYTPTLKNTTAEDKDSNGLTTTGVIKDADNWTLDSGFYKTPKYSLGDYVWYDSNKDG 826

SG11 TPAGYTPTLKNTTAEDKDSNGLTTTGVIKDADNWTLDSGFYKTPKYSLGDYVWYDSNKDG 840

SG04 TPAGYTPTLKNTTAEDKDSNGLTTTGVIKDADNWTLDSGFYKTPKYSLGDYVWYDSNKDG 840

58113 TPAGYTPTLKNTTAEDKDSNGLTTTGVIKDADNWTLDSGFYKTPKYSLGDYVWYDSNKDG 826

********:*****:*********:*******: ********.***.**********:**

**CnaBE3 domain**

SG02 KQDPTEKGIKDVTVTLQNEKGEVIGTTKTDENGKYRFDNLDSGKYKVIFEKPAGLTQTGT 900

MSHR1132_FR821777 KQDPTEKGIKDVTVTLQNEKGEVIGTTKTDENGKYRFDNLDSGKYKVIFEKPAGLTQTGT 900

SG14 KQDPTEKGIKDVTVTLQNEKGEVIGTTKTDENGKYRFDNLDSGKYKVIFEKPAGLTQTGT 900

SG12 KQDPTEKGIKDVTVTLQNEKGEVIGTTKTDENGKYRFDNLDSGKYKVIFEKPAGLTQTGT 900

BN75(CP015758) KQDPTEKGIKDVTVTLQNEKGEVIGTTKTDENGKYRFDNLDSGKYKVIFEKPAGLTQTGT 900

H-EMRSA-15(CP007659) KQDSTEKGIKDVTVTLQNEKGEVIGTTKTDENGKYRFDNLDSGKYKVIFEKPAGLTQTVT 900

SG06 KQDPTEKGITDVTVTLQNEKGEEIGTNSTDDSDTYRFDNLDSGTYSDIFEKPAGLTQTGT 900

DAR4145(CP010526) KQDSTEKGIKDVTVTLQNEKGEVIGTTKTDENGKYRFDNLDSGKYKVIFEKPAGLTQTVT 900

SG03 KQDPTEKGIKDVTVTLQNEKGEVIGTTKTDENGKYRFDNLDSGKYKVIFEKPAGLTQTGT 900

SG07 KQDPTEKGIKDVTVTLQNEKGEVIGTTKTDENGKYRFDNLDSGKYKVIFEKPAGLTQTGT 900

SG13 KQDPTEKGIKDVTVTLQNEKGEVIGTTKTDENGKYRFDNLDSGKYKVIFEKPAGLTQTGT 886

SG11 KQDPTEKGIKDVTVTLQNEKGEVIGTTKTDENGKYRFDNLDSGKYKVIFEKPAGLTQTGT 900

SG04 KQDPTEKGIKDVTVTLQNEKGEVIGTTKTDENGKYRFDNLNSGKYKVIFEKPAGLTQTGT 900

58113 KQDPTEKGIKDVTVTLQNEKGEVIGTTKTDENGKYRFDNLDSGKYKVIFEKPAGLTQTGT 886

*** *****.************ ***..**:...******:**.*. *********** *

**▐→SD repeat region**

SG02 NTTEDDKDADGGEVDVTITDHDDFTLDNGYYEEDTSDSDSDSDSDSDSDSDSDSDSDSDS 960

MSHR1132_FR821777 NTTEDDKDADGGEVDVTITDHDDFTLDNGYYEEDTSDSDSDSDSDSDSDSDSDSDSDSDS 960

SG14 NTTEDDKDADGGEVDVTITDHDDFTLDNGYYEEDTSDSDSDSDSDSDSDSDSDSDSDSDS 960

SG12 NTTEDDKDADGGEVDVTITDHDDFTLDNGYYEEDTSDSDSDSDSDSDSDSDSDSDSDSDS 960

BN75(CP015758) NTTEDDKDADGGEVDVTITDHDDFTLDNGYYEEDTSDSDSDSDSDSDSDSDSDSDSDSDS 960

H-EMRSA-15(CP007659) NTTEDDKDADGGEVDVTITDHDDFTLDNGYFEEDTSDSDSDSDSDSDSDSDSDSDSDSDS 960

SG06 NTTEDDKDADGGEVDVTITDHDDFTLDNGYYEEDTSDSDSDSDSDSDSDSDSDSDSDSDS 960

DAR4145(CP010526) NTTEDDKDADGGEVDVTITDHDDFTLDNGYYEEETSDSDSDSDSDSDSDSDSDSDSDSDS 960

SG03 NTTEDDKDADGGEVDVTITDHDDFTLDNGYYEEDTSDSDSDSDSDSDSDSDSDSDSDSDS 960

SG07 NTTEDVKDADGGEVDVTITDHDDFTLDNGYYEEDTSDSDSDSDSDSDSDSDSDSDSDSDS 960

SG13 NTTEDDKDADGGEVDVTITDHDDFTLDNGYYEEDTSDSDSDSDSDSDSDSDSDSDSDSDS 946

SG11 NTTEDDKDADGGEVDVTITDHDDFTLDNGYYEEDTSDSDSDSDSDSDSDSDSDSDSDSDS 960

SG04 NTTEDDKDADGGEVDVTITDHDDFTLDNGYYEEDTSDSDSDSDSDSDSDSDSDSDSDSDS 960

58113 NTTEDDKDADGGEVDVTITDHDDFTLDNGYYEEDTSDSDSDSDSDSDSDSDSDSDSDSDS 946

***** ************************:**:**************************

SG02 DSDSDSDSDSDSDSDSDSDSDSDSDSDSDSDSDSDSDS--------DSDSD--SDSDSDS 1010

MSHR1132_FR821777 DSDSDSDSDSDSDSDSDSDSDSDSDSDSDSDSDSDSDSDS------DSDSD--SDSDSDS 1012

SG14 DSDSDSDSDSDSDSDSDSDSDSDSDSDSDSDSDSDSDSDSDSDSDSDSDSDSDSDSDSDS 1020

SG12 DSDSDSDSDSDSDSDSDSDSDSDSDSDSDSDSDSDSDSDSDSDSDSDSDSDSDSDSDSDS 1020

BN75(CP015758) DSDSDSDSDSDSDSDSDSDSDSDSDSDSDSD----------S--DSDS--DSDS--DSDS 1004

H-EMRSA-15(CP007659) DSDSDSDSDSDSDSDSDSDSDSDSDSDSDSDSDSD------SD----SDSD----SDSD- 1005

SG06 DSDSDSDSDSDSDSDSDSDSDSDSDSDSDSDSDSDSDSDSD------SDSD----SDSD- 1009

DAR4145(CP010526) DSDSDSDSDSDSDSDSDSDSDSDSDSDSDSDSDSDSDSDSD------SDSD----SDSD- 1009

SG03 DSDSDSDSDSDSDSDSDSDSDSDSDSDSDSD--------SD--------SD----SD--- 997

SG07 DSDSDSDSDSDSDSDSDSDSDSDSDSDSDSD--------SD--------SD----SD--- 997

SG13 DSDSDSDSDSDSDSDSDSDSDSDSDSDSDSDSDSDSDSDSDSDSDSDSDSD----SD--- 999

SG11 DSDSDSDSDSDSDSDSDSDSDSDSDSDSDSDSDSDSDSDSDSDSDSDSDSD----SDSDS 1016

SG04 DSDSDSDSDSDSDSDSDSDSDSDSDSDSDSDSDSDSDSDSDSDSDSDSDSD----SDSDS 1016

58113 DSDSDSDSDSDSDSDSDSDSDSDSDSDSDSDSDSDSDSDSDSDSDSDSDSD----SDSDS 1002

******************************* * *

SG02 --DSDSDSDS--DSDSDSDS--DSDSDSD--SDSDSDSD--SDSDSDS--DSDSDSD--- 1055

MSHR1132_FR821777 --DSDSDSDS--DSDSDSDS--DSDSDSD--SDSDSDSD--SDSDSDS--DSDSD----- 1055

SG14 DSDSDSDSDSDSDSDSDSDSDSDSDSDSDSDSDSDSDSDSDSDSDSDSDSDSDSDSDSDS 1080

SG12 DSDSDSDSDSDSDSDSDSDSDSDSDSDSDSDSDSDSDSDSDSDSDSDSDSDSDSDSDSDS 1080

BN75(CP015758) --DSDS--DSDS--DSDS--DSDS--DSDS--DSDS--DSDS--DSDS--DSDSD--SDS 1044

H-EMRSA-15(CP007659) ---SDSDSDS--DSD--SDS--DSDSDSDS--DSDS--D--S--DSDS--DSDSD----- 1041

SG06 ---SDSDSDS--DSD--SDS--DSDSDSDS--DSDS--D--SDSDSDS--DSDSDSDS-- 1050

DAR4145(CP010526) ---SDSDSDS--DSD----S--DSDSDSDS--DSDS--D--SDSDSDS--DSDSDSDS-- 1048

SG03 ---SD--------------S--D--SDSD----SDS--D--S--DS----------DS-- 1014

SG07 ---SDSDS----DSD----S--D--SDSD----SDS--D--S--DS----------DS-- 1020

SG13 ---SDSDSDSDSDSD--SDS--DSDSDSDS--DSDS--D--SDSDSDSDSDSDSDSDSDS 1046

SG11 --DSDSDSDSDSDSDSESDS--DSDSDSDSDSDSDS--D--SDSDSDSDSDSDSDSDSDS 1068

SG04 --DSDSDSDSDSDSDSDSDS--DSDSDSDSDSDSDS--D--SDSDSDSDSDSDSDSDSDS 1068

58113 --DSDSDSDSDSDSDSDSDS--DSDSDSDSDSDSDS--D--SDSDSDSDSDSDSDSDSDS 1054

** * *** *** * * **

SG02 -----SDSDSDSDSDSDSDSDSDSDSDSDSDSDSDSDSDSDSDSDSDSDSDSDSDSDSDS 1110

MSHR1132_FR821777 -SDSDSDSDSDSDSDSDSDSDSDSDSDSDSDSDSDSDSDSDSDSDSDSDSDSDSDSDSDS 1114

SG14 DSDSDSDSDSDSDSDSDSDSDSDSDSDSDSDSDSDSDSDSDSDSDSDSDSDSDSDSDSDS 1140

SG12 DSDSDSDSDSDSDSDSDSDSDSDSDSDSDSDSDSDSDSDSDSDSDSDSDSDSDSDSDSDS 1140

BN75(CP015758) E—RKS 1048

H-EMRSA-15(CP007659) ---------------SDSDSDSDSDSDSDSDSDSDSDSDSDSDSDSDSDSDSDSDSDSDS 1086

SG06 ------------DSDSDSDSDSDSDSDSDSDSDSDSDSDSDSDSDSDSDSDSDSDNDSDS 1098

DAR4145(CP010526) ------------DSDSDSDSDSDSDSDSDSDSDSDSDSDSDSDSDSDSDSDSDSDSDSDS 1096

SG03 ------------DSDSDSDSDSDSDSDSDSDSDSDSDSDSDSDSDSDSDSDSDSDNDSDS 1062

SG07 ------------DSDSDSDSDSDSDSDSDSDSDSDSDSDSDSDSDSDSDSDSDSDNDSDS 1068

SG13 ----------DSDSDSDSDSDSDSDSDSDSDSDSDSDSDSDSDSDSDSDSDSDSDSDSDS 1096

SG11 DSDS------DSDSDSDSDSDSDSDSDSDSDSDSDSDSDSDSDSDSDSDSDSDSDSDSDS 1122

SG04 DSDSDSTQIAIQDSDSDSDSDSDSDSDSDSDSDSDSDSDSDSDSDSDSDSDSDSDSDSDS 1128

58113 DSDS------DSDSDSDSDSDSDSDSDSDSDSDSDSDSDSDSDSDSDSDSDSDSDSDSDS 1108

▐ **→ W region**  **LPXTG motif** ▐ **→ W region**

SG02 DSDSDSDAGKHTPAKPMSTVKDHHNTAKALPETGSEDNGTNNATLFGGLFAALGSLLLFG 1170

MSHR1132_FR821777 DNDSDSDAGKHTPAKPMSTVKDHHNKAKALPETGSEDNGTNNATLFGGLFAALGSLLLFG 1174

SG14 DSDSDSDAGKHTPAKPMSTVKDHHNTAKALPETGSEDNGTNNATLFGGLFAALGSLLLFG 1200

SG12 DSDSDSDAGKHTPAKPMSTVKDHHNTAKALPETGSEDNGTNNATLFGGLFAALGSLLLFG 1200

BN75(CP015758) 1048

H-EMRSA-15(CP007659) DSDSDSDAGKHTPVKPMSTTKDHHNKAKALPETGSENNGSNNATLFGGLFAALGSLLLFG 1146

SG06 DSDSDSDAGKHTPAKPMSTVKDHHNKAKALPETGSEDNGSNNATLFGGLFAALGSLLLFG 1158

DAR4145(CP010526) DSDSDSDAGKHTPVKPMSTTKDHHNKAKALPETGSENNGSNNATLFGGLFAALGSLLLFG 1156

SG03 DSDSDSDAGKHTPAKPMSTVKDHHNKAKALPETGSEDNGTNNATLFGGLFAALGSLLLFG 1122

SG07 DSDSDSDAGKHTPAKPMSTVKDHHNKAKALPETGSEDNGTNNATLFGGLFAALGSLLLFG 1128

SG13 DSDSDSDAGKHTSAKPMSTVKDHHNKAKALPETGSEDNGSNNATLFGGLFAALGSLLLFG 1156

SG11 DSDSDSDAGKHTSAKPMSTVKDHHNKAKALPETGSEDNGSNNATLFGGLFAALGSLLLFG 1182

SG04 DSDSDSDAGKHTSAKPMSTVKDHHNKAKALPETGSEDNGSNNATLFGGLFAALGSLLLFG 1188

58113 DSDSDSDAGKHTSAKPMSTVKDHHNKAKALPETGSEDNGSNNATLFGGLFAALGSLLLFG 1168

SG02 RRKKQDK 1177

MSHR1132_FR821777 RRKKQDK 1181

SG14 RRKKQDK 1207

SG12 RRKKQDK 1207

BN75(CP015758) 1048

H-EMRSA-15(CP007659) RRKKQNK 1153

SG06 RRKKQDK 1165

DAR4145(CP010526) RRKKQNK 1163

SG03 RRKKQDK 1129

SG07 RRKKQDK 1135

SG13 RRKKQDK 1163

SG11 RRKKQDK 1189

SG04 RRKKQDK 1195

58113 RRKKQDK 1175
