# Supplementary material for: Molecular Epidemiological Characterization of Staphylococcus argenteus Clinical Isolates in Japan: Identification of Three Clones (ST1223, ST2198, and ST2550) and a Novel Staphylocoagulase Genotype XV
Source: Microorganisms. 2019 Sep 24;7(10):389. doi: 10.3390/microorganisms7100389 (PMC6843175; doi:10.3390/microorganisms7100389)
Supplement: Supplementary file 1 [file microorganisms-07-00389-s001.zip › Supplementary materials R1/Supple Fig-S1-R1a.pptx]

## Slide 1
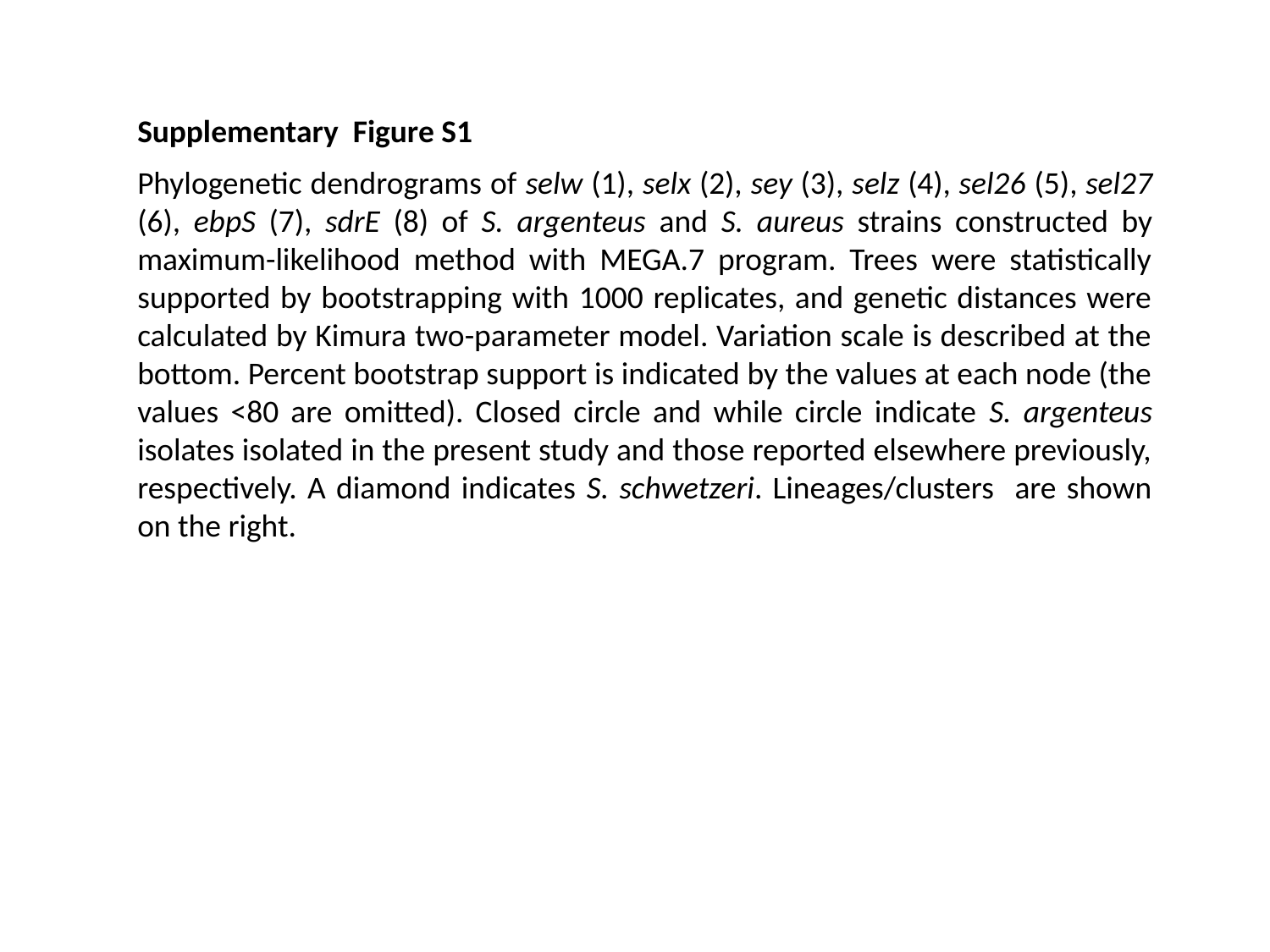

Supplementary Figure S1
Phylogenetic dendrograms of selw (1), selx (2), sey (3), selz (4), sel26 (5), sel27 (6), ebpS (7), sdrE (8) of S. argenteus and S. aureus strains constructed by maximum-likelihood method with MEGA.7 program. Trees were statistically supported by bootstrapping with 1000 replicates, and genetic distances were calculated by Kimura two-parameter model. Variation scale is described at the bottom. Percent bootstrap support is indicated by the values at each node (the values <80 are omitted). Closed circle and while circle indicate S. argenteus isolates isolated in the present study and those reported elsewhere previously, respectively. A diamond indicates S. schwetzeri. Lineages/clusters are shown on the right.

## Slide 2
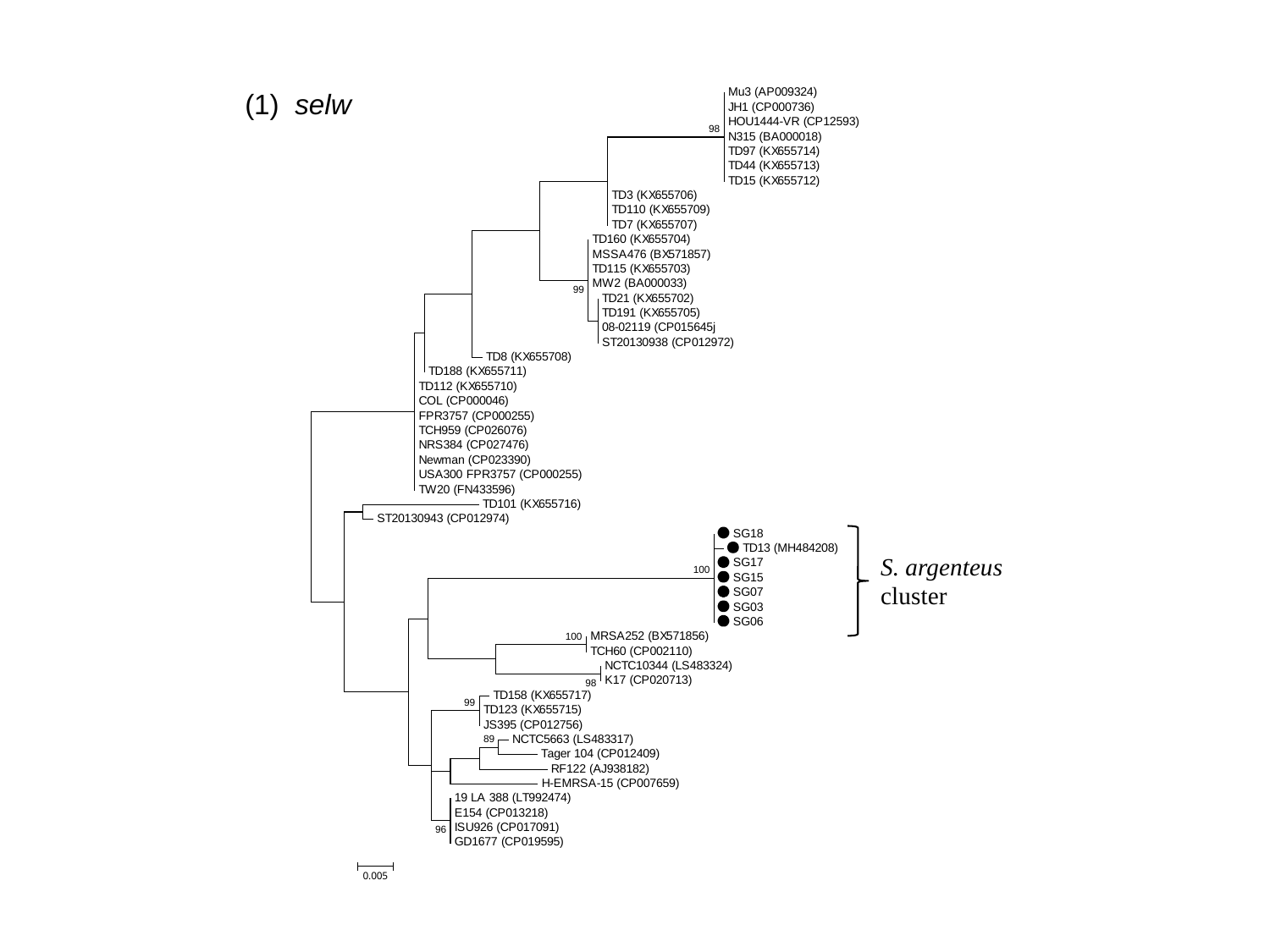

(1) selw
S. argenteus cluster

## Slide 3
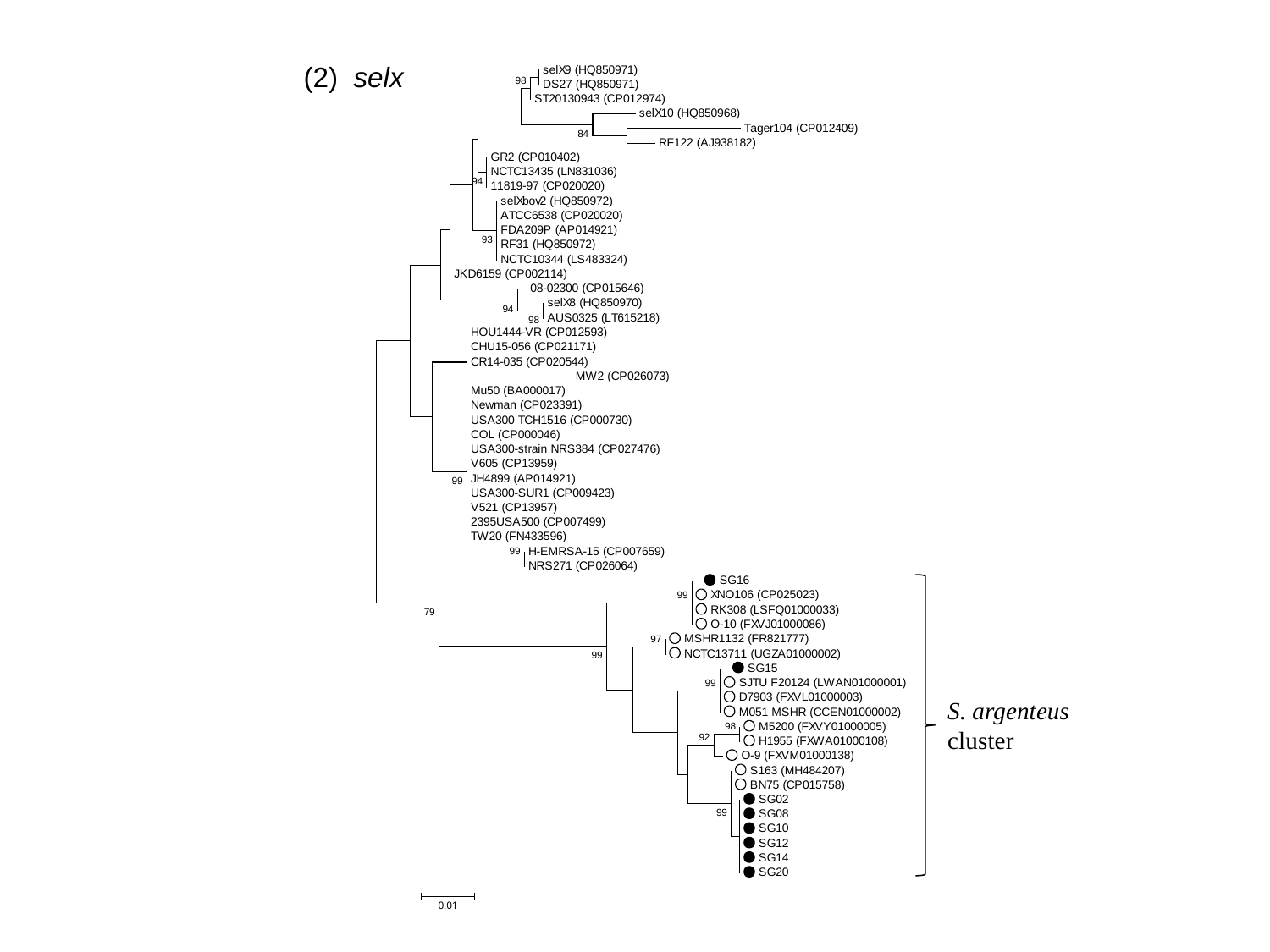

(2) selx
S. argenteus cluster

## Slide 4
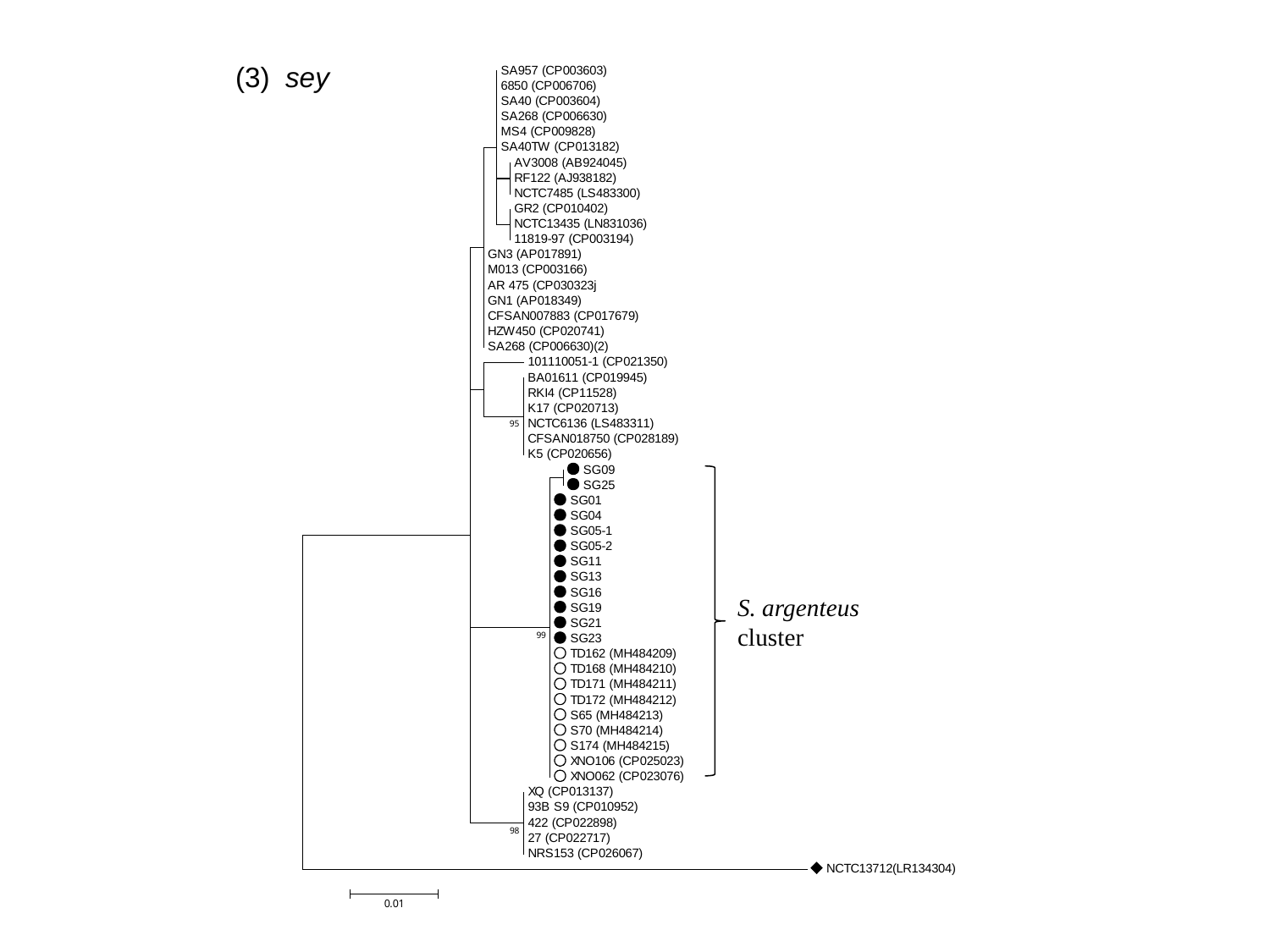

(3) sey
S. argenteus cluster

## Slide 5
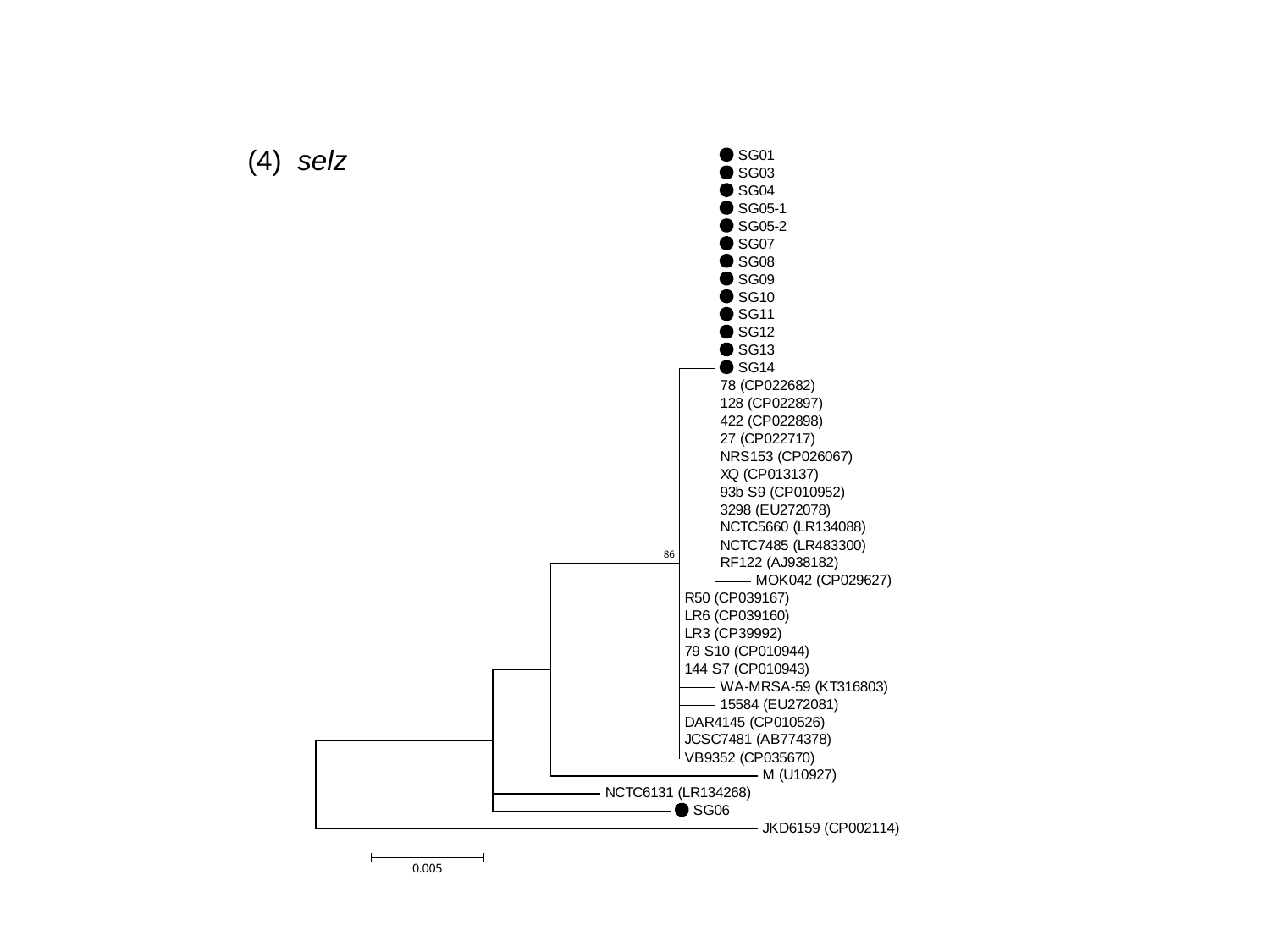

(4) selz

## Slide 6
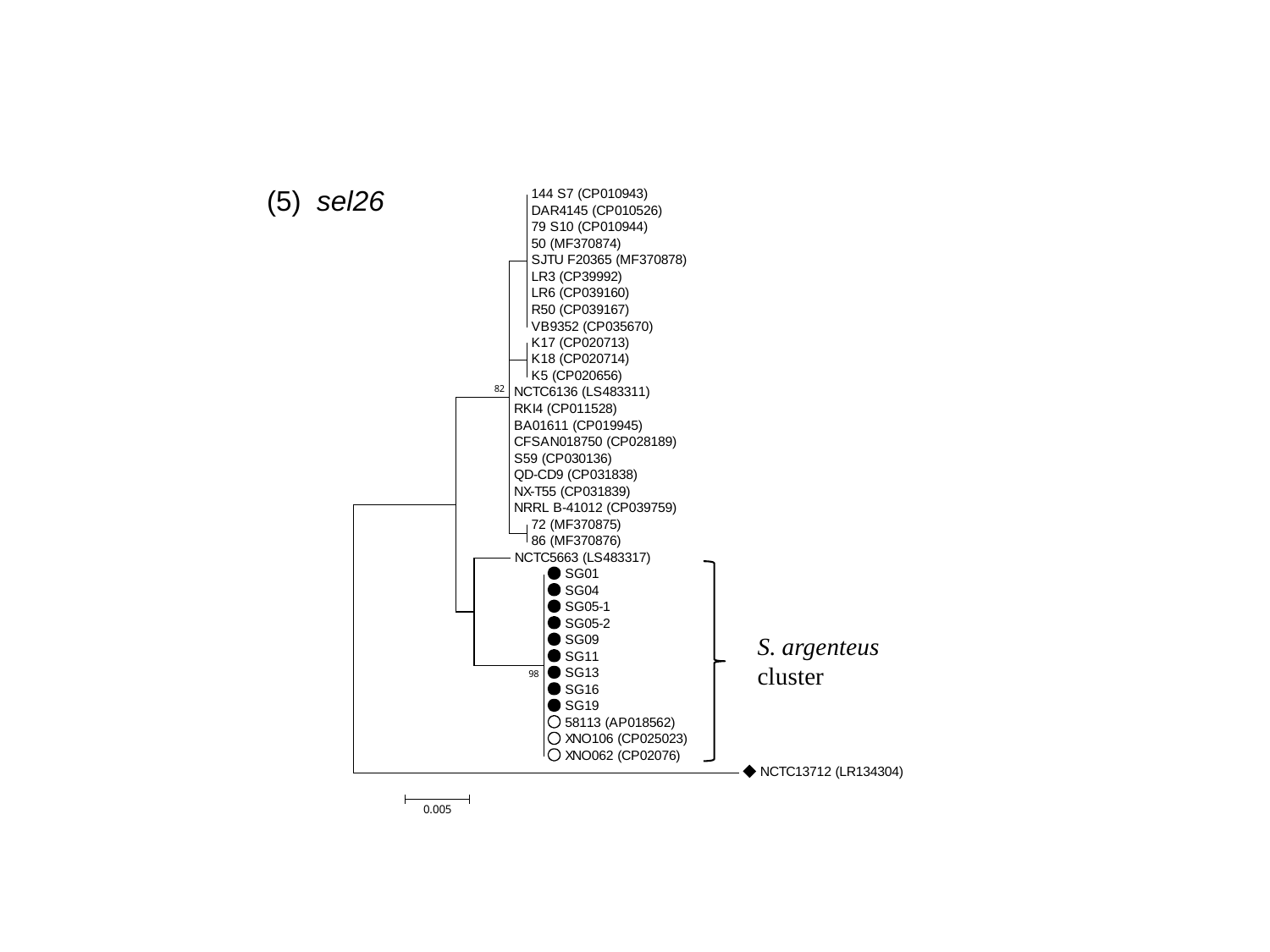

(5) sel26
S. argenteus cluster

## Slide 7
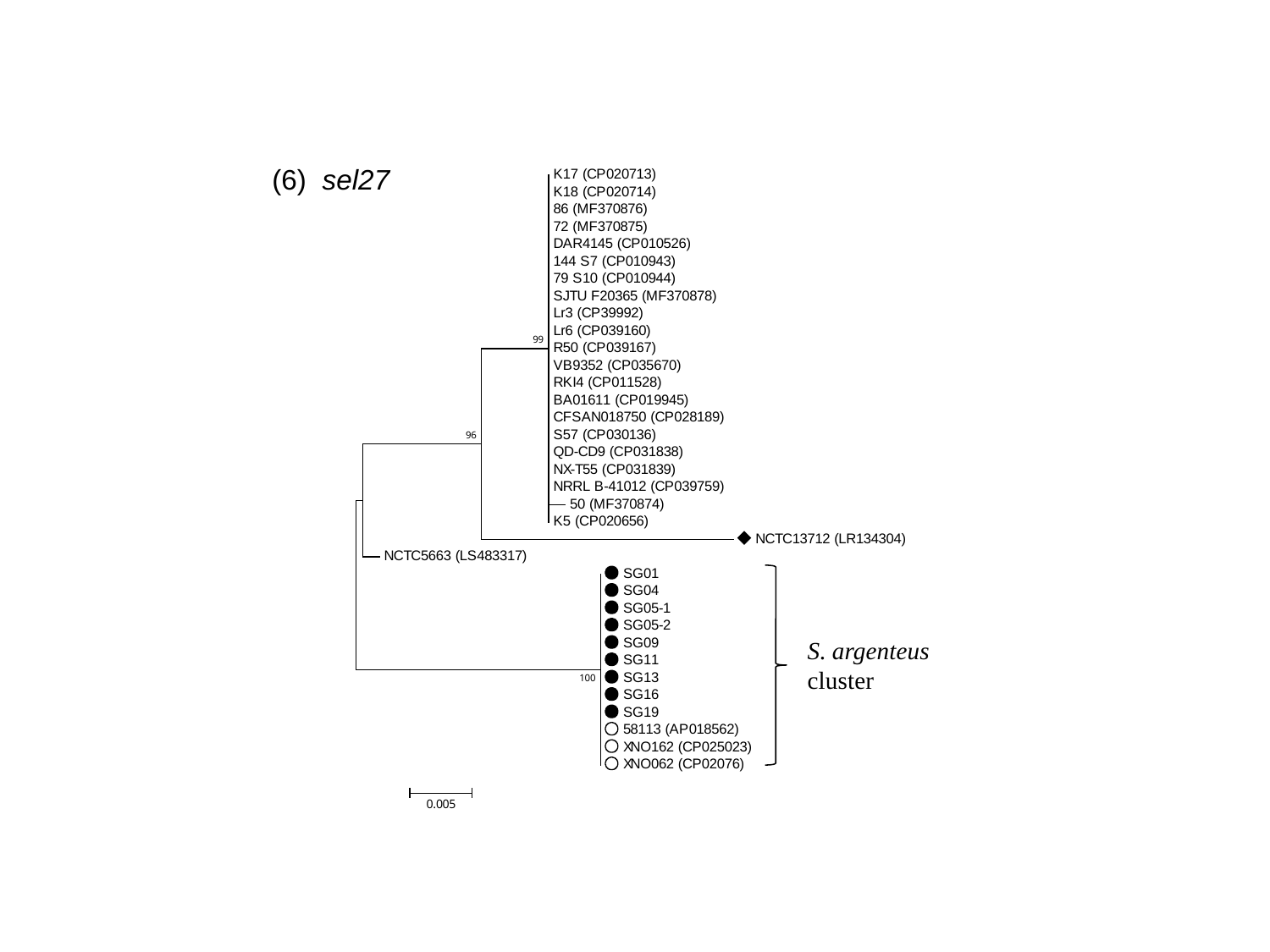

(6) sel27
S. argenteus cluster

## Slide 8
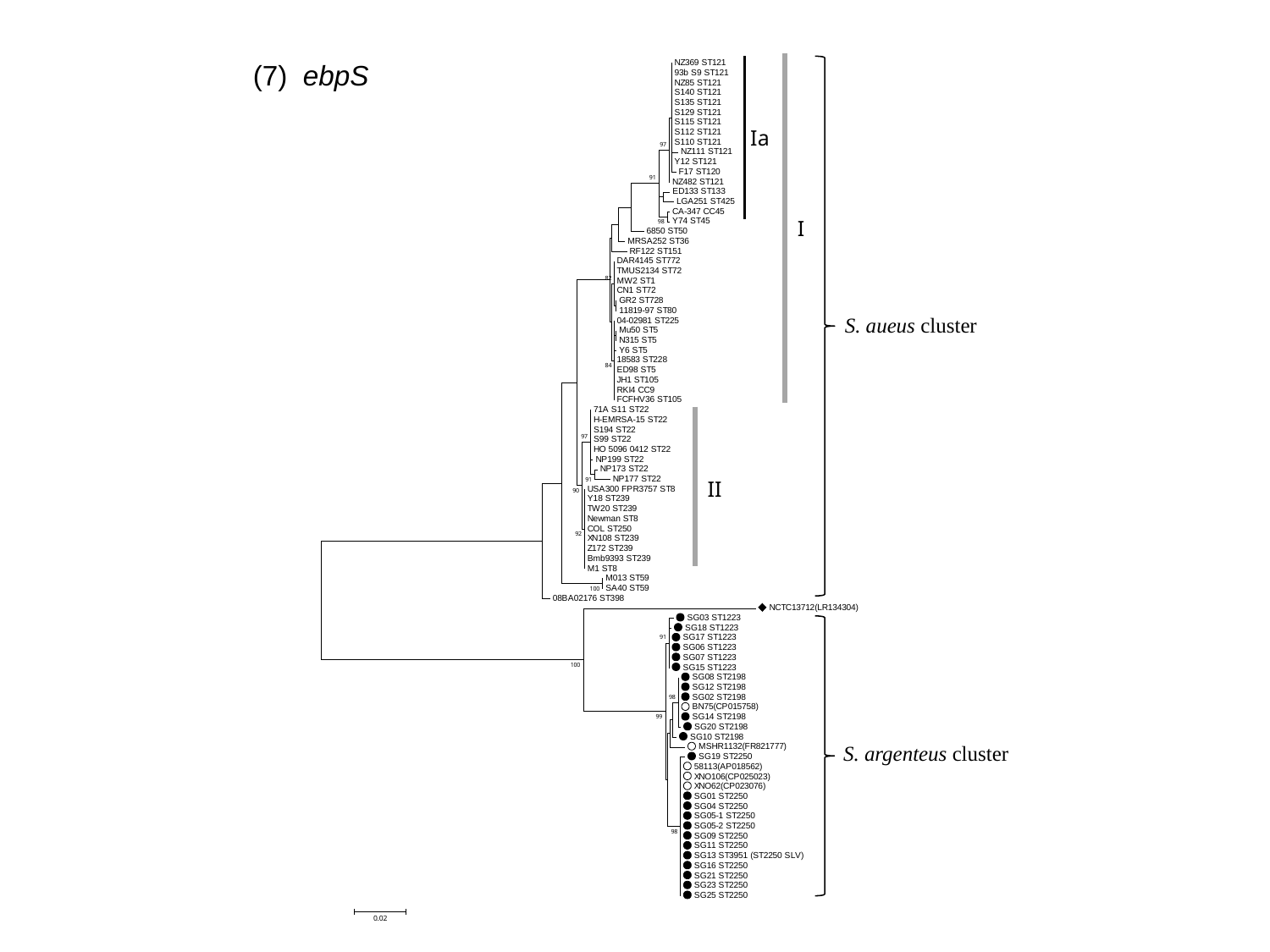

(7) ebpS
Ia
I
S. aueus cluster
II
S. argenteus cluster

## Slide 9
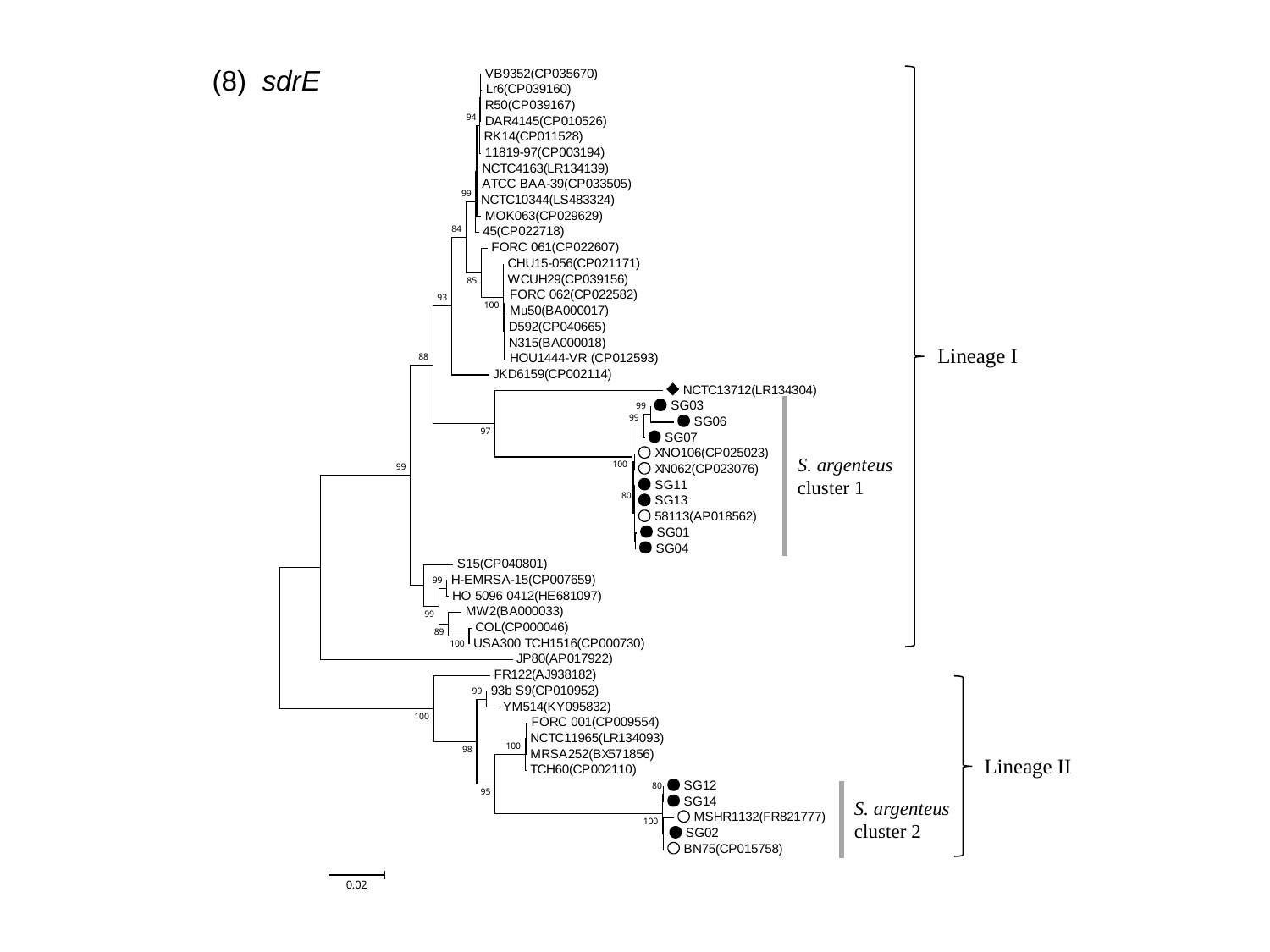

(8) sdrE
Lineage I
S. argenteus cluster 1
Lineage II
S. argenteus cluster 2
